# Upregulated WDR5 promotes proliferation, self-renewal and chemoresistance in bladder cancer via mediating H3K4 trimethylation

**DOI:** 10.1038/srep08293

**Published:** 2015-02-06

**Authors:** Xu Chen, Weibin Xie, Peng Gu, Qingqing Cai, Bo Wang, Yun Xie, Wen Dong, Wang He, Guangzheng Zhong, Tianxin Lin, Jian Huang

**Affiliations:** 1 Department of Urology, Sun Yat-sen Memorial Hospital, Sun Yat-sen University, Guangzhou, China; 2 Guangdong Provincial Key Laboratory of Malignant Tumor Epigenetics and Gene Regulation, Sun Yat-sen Memorial Hospital, Sun Yat-sen University, Guangzhou, China; 3 Department of Internal Medicine, Sun Yat-sen University Cancer Center, Guangzhou, China

## Abstract

WD repeat domain 5 (WDR5) plays an important role in various biological functions through the epigenetic regulation of gene transcription; however, its role in bladder cancer remains largely unknown. Our study investigated the role of WDR5 in bladder cancer and demonstrated that WDR5 was upregulated in bladder cancer tissues, and elevated WDR5 protein levels positively correlated with advanced tumor stage and poor survival. Through gain or loss of function, we demonstrated that WDR5 promoted proliferation, self-renewal and chemoresistance to cisplatin in bladder cancer cells *in vitro*, and tumor growth *in vivo*. Mechanistically, WDR5 regulated various functions in bladder cancer by mediating the transcription of cyclin B1, cyclin E1, cyclin E2, UHMK1, MCL1, BIRC3 and Nanog by histone H3 lysine 4 trimethylation. Therefore, we have discovered that WDR5 plays an important role in bladder cancer suggesting that WDR5 is a potential biomarker and a promising target in the treatment of bladder cancer.

Bladder cancer is one of the most common cancers, causing approximately 150,000 deaths per year worldwide^1^. It is a clinically heterogeneous disease: noninvasive lesions that usually recur but rarely progress, and aggressive muscle-invasive lesions that progress and are associated with poor long-term survival^2^. Emerging evidence shows that the mechanisms of bladder cancer are complex, which may include the alteration of genomic regions^3^ and epigenetic modification^4^,^5^,^6^ in urothelial cells. Despite recent progress, the molecular mechanisms underlying bladder cancer pathogenesis remain to be further elucidated.

Chromatin modifications, including histone methylation, acetylation, phosphorylation, ubiquitylation and ADP ribosylation, have been found to play crucial roles in gene expression^7^. Regarding histone methylation, the Polycomb repression complex 2 (PRC2) mediates histone H3 lysine 27 trimethylation (H3K27me3), which is correlated with gene repression, whereas the MLL/SET1 complexes mediates H3K4 methylation, which is correlated with gene activation^8^. WDR5 is a core component of the MLL/SET1 complexes and exerts methyltransferase activity^9^,^10^. The present study reveals that WDR5 plays a critical role in embryonic stem cell self-renewal by interacting with Oct4 and mediating H3K4 methylation^11^. In addition, WDR5 induced by hypoxia interacts with HDAC3 to activate mesenchymal gene expression and promotes epithelial-mesenchymal transition by H3K4 methylation^12^. Moreover, WDR5 is overexpressed in prostate cancer and promotes proliferation upon androgen stimulation^13^. A previous study revealed that WDR5 silencing attenuates oncogene ErbB2 overexpression in breast cancer by decreasing H3K4me3 levels on its promoter^14^. Although basic knowledge related to WDR5 has increased recently, the expression pattern and biological function of WDR5 in bladder cancer remains largely unknown.

In this study, we found that WDR5 expression was increased in bladder cancer tissues and correlated with advanced tumor stage and poor survival. Furthermore, WDR5 promoted bladder cell proliferation, self-renewal and chemoresistance to cisplatin *in vitro*, and accelerated tumor growth *in vivo*. We identified some target genes of WDR5 by microarray and chromatin immunoprecipitation (ChIP) qPCR.

## Results

### WDR5 is upregulated in bladder cancer tissues

**Figure 1:**
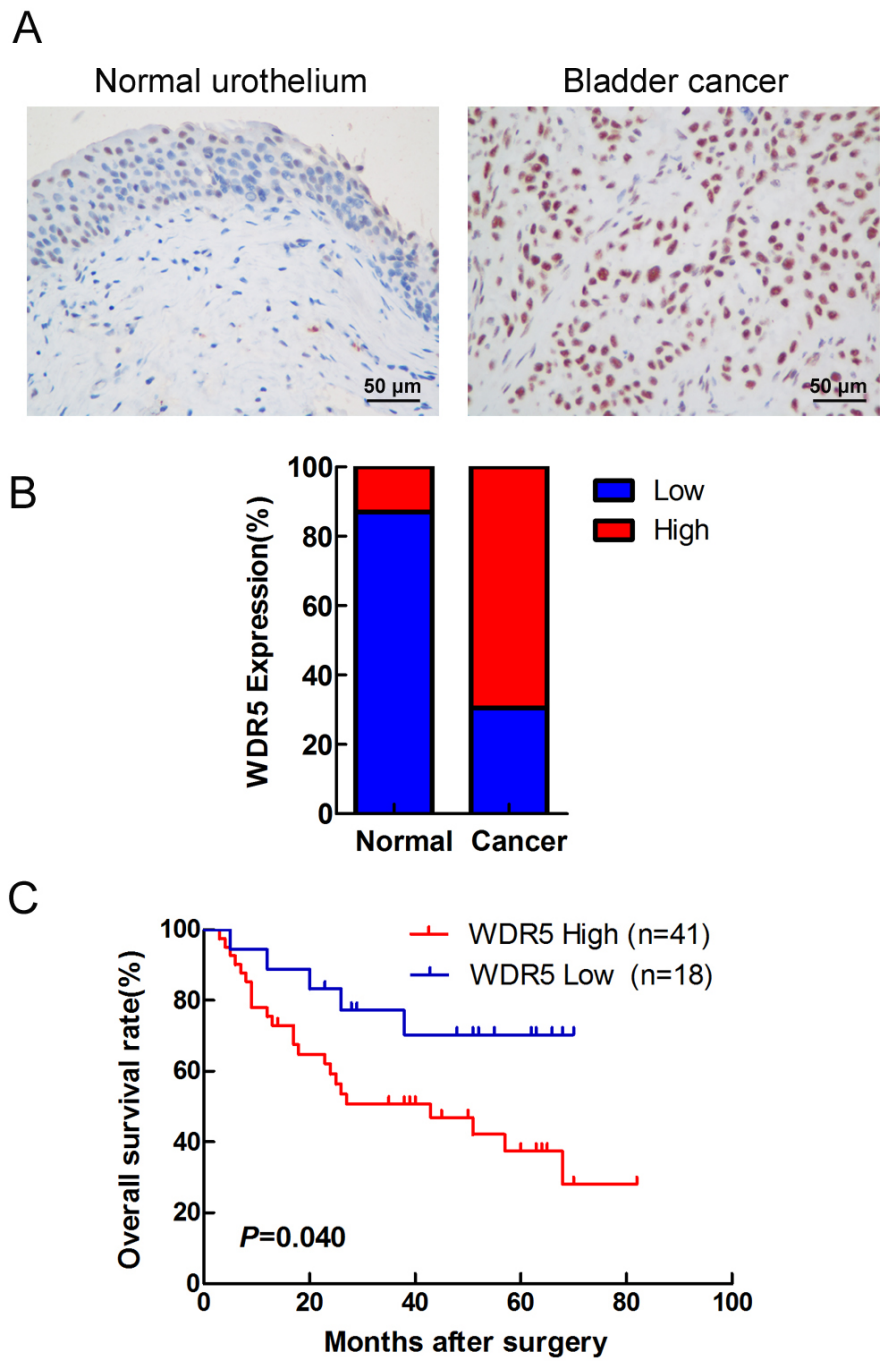
WDR5 was upregulated in bladder cancer tissues. (A) The expression of WDR5 was detected in bladder normal urothelium and cancer tissue by IHC, and representative samples are shown at 400× magnification. (B) The expression of WDR5 was quantified by H-score in normal urothelium and bladder cancer. The samples were classed as low (H-score <200) or high (H-score ≥200) WDR5 expression. (C) The overall survival rates of the 59 bladder cancer patients were compared in the WDR5-low and WDR5-high groups. Statistical significance was determined using the log-rank test.

To detect the expression of WDR5 in bladder cancer, we examined WDR5 expression in 134 bladder cancer tissues and 77 normal tissues by immunohistochemistry (IHC). The positive and negative controls of WDR5 in IHC were shown in Supplemental Figure 1. WDR5 was obviously upregulated in bladder cancer tissues compared with normal tissues (Fig. 1A). Most of the bladder cancer tissues (69.4%, 93/134 cases) were found to exhibit high WDR5 expression. By contrast, most of the normal tissues (87%, 67/77 cases) expressed low WDR5 (Fig. 1B). The log-rank test revealed that WDR5 expression correlated significantly with the overall survival of bladder cancer patients (Fig. 1C). The overall survival for bladder cancer patients with higher WDR5 expression was significantly shorter than those patients with low WDR5 expression (p = 0.04). Upon clinicopathological correlation analysis, elevated WDR5 protein levels positively correlated with advanced tumor stage of bladder cancer (Table 1, p = 0.005).

### WDR5 promotes bladder cancer cell proliferation by regulating the cell cycle

**Figure 2:**
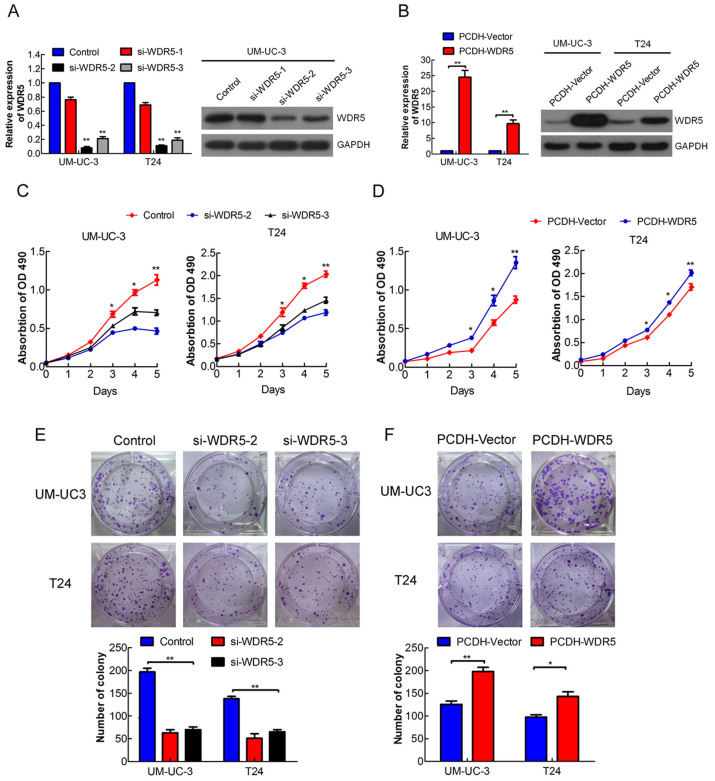
WDR5 promotes bladder cancer cell proliferation *in vitro*. (A) Efficiency of WDR5 knockdown in UM-UC-3 cells by siRNA was verified by qRT-PCR and Western blotting. (B) Efficiency of WDR5 stable overexpression in UM-UC-3 and T24 cells by lentivirus was verified by qRT-PCR and Western blotting. The samples were derived from the same experiment and that blots were processed in parallel. The full-length blots are presented in Supplementary Figure 4. (C and D) Influence of WDR5 knockdown or overexpression on viability of UM-UC-3 and T24 cells was measured by MTT assay. (E and F) Effect of WDR5 knockdown or overexpression on colony formation was measured in UM-UC-3 and T24 cells. Representative images of the colonies, stained with crystal violet, were shown above, whereas the colony counts were shown below the graph. Three independent experiments yielded similar results. The results are presented as the means ± SD of values obtained in three independent experiments. Statistical significance was calculated using the Student's t-tests when only two groups were compared or ANOVA when more than two groups were compared. *p < 0.05, **p < 0.01.

To study the role of WDR5 in bladder cancer, we created a WDR5 gain or loss of function in bladder cancer cells. As shown in Figure 2A, WDR5 was remarkably downregulated in UM-UC-3 cells by the siRNA si-WDR5-2 or si-WDR5-3, but not si-WDR5-1. WDR5 was observably upregulated in UM-UC-3 and T24 cells through lentiviral infection (Fig. 2B). Moreover, WDR5 knockdown significantly reduced viability and colony formation of UM-UC-3 and T24 cells (Fig. 2C and E, p < 0.05). In contrast, overexpression of WDR5 enhanced bladder cancer cell viability and colony formation (Fig. 2D and F). Therefore, these data suggest that WDR5 promotes the proliferation of bladder cancer cells *in vitro*.

**Figure 3:**
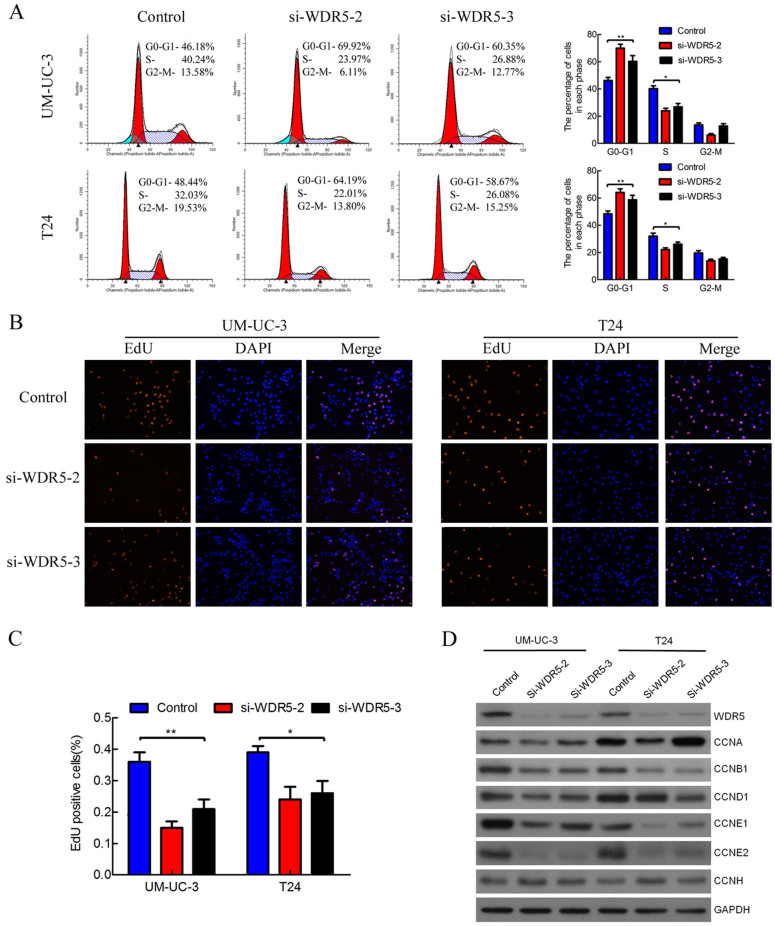
WDR5 knockdown induced G0/G1 phase cell cycle arrest in bladder cancer cells. (A) UM-UC-3 and T24 cells were transfected with WDR5 or control siRNA for 48 h and analyzed by flow cytometry. Percentages (%) of cell populations at different stages of cell cycles are listed within the panels. All histograms show the percentage (%) of cell populations from three independent experiments. (B) The cell population at S phase was measured by EdU assay. Blue color represents the nucleus and red color indicates S phase cells (EdU-positive). (C) Histological analysis of the percentage of EdU-positive cells in control and WDR5 knockdown. (D) Effect of WDR5 knockdown on regulation of cyclins was detected by Western blotting. WDR5 silencing reduced the expression of cyclin B1, cyclin E1 and cyclin E2. The samples were derived from the same experiment and that blots were processed in parallel. The full-length blots are presented in Supplementary Figure 4.The results are presented as the means ± SD of values obtained in three independent experiments. Statistical significance was calculated using the ANOVA. *p < 0.05, **p < 0.01.

Next, we performed flow cytometry and EdU assays to characterize whether WDR5 was involved in the cell cycle. Interestingly, WDR5 silencing dramatically increased the cell population at G0/G1 phase, whereas it reduced the cell population at S and G2/M phase (Fig. 3A). Moreover, WDR5 knockdown significantly decreased the cell population at S phase in UM-UC-3 and T24 cells by EdU assay (Fig. 3B-C, p < 0.05). Given that WDR5 knockdown induced cell cycle arrest, we examined the effect of knockdown on cyclins. Interestingly, WDR5 silencing obviously reduced expression of cyclin B1, E1 and E2, whereas cyclin A, D1 and H were mostly unaffected (Fig. 3D). Collectively, these results demonstrate that WDR5 knockdown mainly induces G0/G1 phase cell cycle arrest in bladder cancer cells.

### WDR5 plays an important role in bladder cancer cell self-renewal

**Figure 4:**
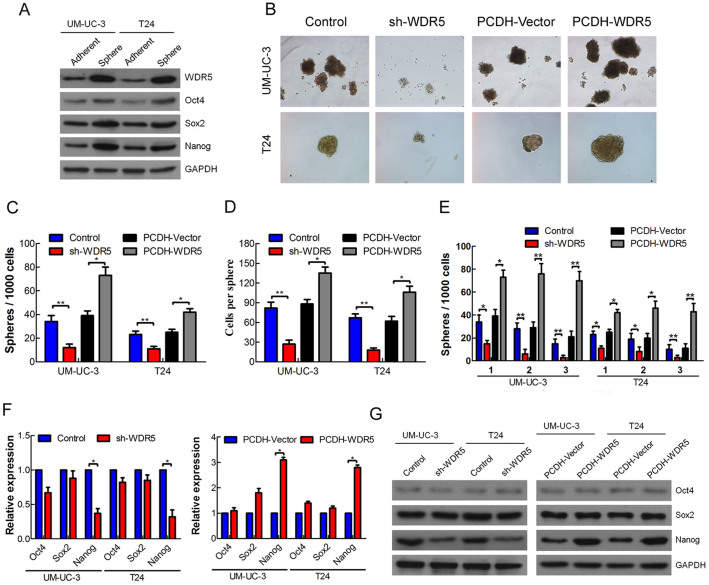
WDR5 plays an important role in bladder cancer cell self-renewal. (A) The expression of WDR5, Oct4, Sox2 and Nanog was detected in UM-UC-3 and T24 sphere and adherent cells by Western blotting. (B) The self-renewal capacity of UM-UC-3 and T24 cells after stable transfection was measured by spheres formation. (C and D) The number of spheres reflected the quantity of cells capable of *in vitro* self-renewal, whereas the number of cells/sphere measured the self-renewal capacity of each sphere-generating cell. (E) The self-renewal capacity was further investigated by serial passage assay. (F and G) The expression of pluripotency transcription factors Oct4, Sox2 and Nanog in WDR5 knockdown or WDR5 overexpression cells was detected by qRT-PCR and Western blotting. The samples were derived from the same experiment and that blots were processed in parallel. The full-length blots are presented in Supplementary Figure 4.The results are presented as the means ± SD of values obtained in three independent experiments. Statistical significance was calculated using the Student's t-tests. *p < 0.05, **p < 0.01.

Although WDR5 involves in the self-renewal of embryonic stem cells^15^, whether WDR5 regulates the self-renewal of cancer stem cells remained largely unknown. To address this, we first performed the sphere culture in UM-UC-3 and T24, and detected the expression of pluripotency transcription factors in the sphere and adherent cells. We found the Oct4, Sox2 and Nanog were obviously upregulated in sphere cells compared with adherent cells, suggesting that the UM-UC-3 and T24 spheres had the characteristics of cancer stem cells (Fig. 4A). Interestingly, we found that WDR5 was aslo upregulated in UM-UC-3 and T24 spheres, suggesting that WDR5 might associate with cancer stem cells (Fig. 4A). So we stably knockdown WDR5 in UM-UC3 and T24 cells by lentivirus (Fig. S2). The number and size of the spheres was remarkably decreased in the WDR5 knockdown UM-UC3 and T24 cells, but increased in the WDR5 overexpressed UM-UC3 and T24 cells (Fig. 4B–D). Furthermore, knockdown of WDR5 surprisingly attenuated, whereas overexpression of WDR5 well maintained, the ability of sphere formation in the serial passage assay (Fig. 4E). In addition, WDR5 silencing mainly reduced, but overexpression of WDR5 increased, the expression of Nanog (Fig. 4F–G). Overexpression of Nanog remarkably increased the number and size of the spheres in UM-UC3 and T24 cells, suggesting that Nanog promoted the self-renewal of bladder cancer cells (Fig. S3). Therefore, these data suggest that WDR5 is associated with CSCs and may regulate bladder cancer cells self-renewal by mediating Nanog.

### WDR5 enhances bladder cancer cells chemoresistance to cisplatin

**Figure 5:**
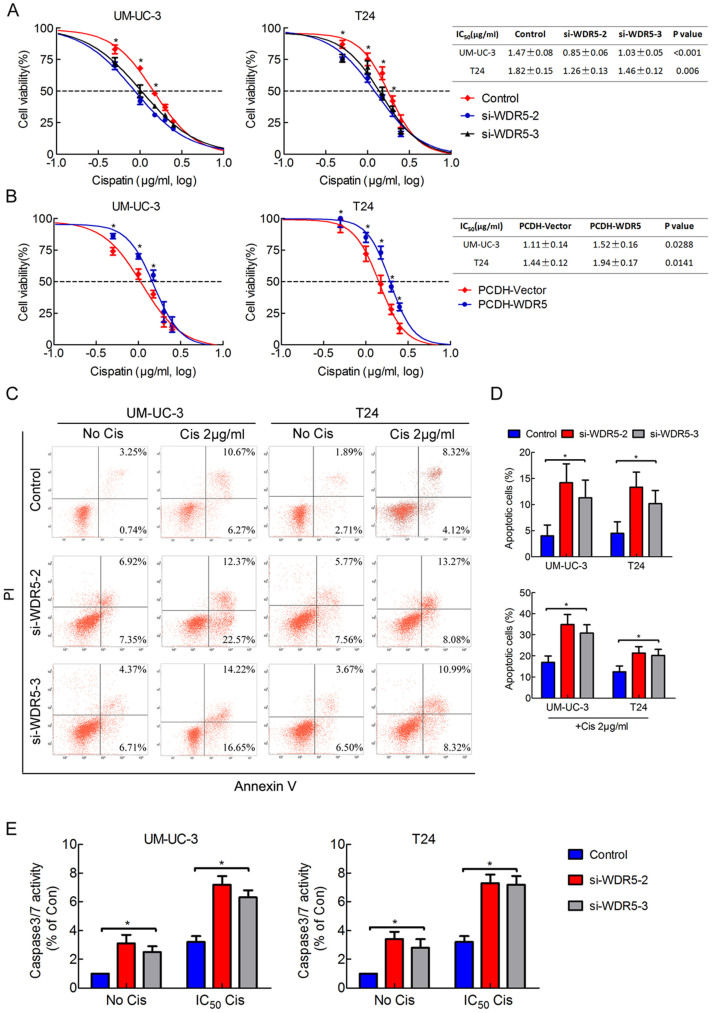
WDR5 enhances bladder cancer cell chemoresistance to cisplatin. (A) The cell viability of cells transfected with WDR5 or control siRNA and treated with cisplatin for 48 h was analyzed by MTT assay. (B) The cell viability of cells that stably overexpressed WDR5 or control vector and treated with cisplatin for 48 h was analyzed by MTT assay. For calculation of IC_50_, four parameter logistic curve (best-fit solution, nonlinear regressiondynamic fitting) and normality tests are used (Graph Pad Prism 5). (C) The cells 24 h after transfection with control or WDR5 siRNA were treated with 0 or 2 μg/ml cisplatin for 24 h. The percentage of apoptotic cells was analyzed by flow cytometer. (D) The histogram showed the percentage (%) of late and early apoptotic cells from three independent experiments. (E) Caspase 3/7 activity assay was performed on the UM-UC-3 and T24 cells transfected with control or WDR5 siRNA and treated with or without IC_50_ concentration of parental cells cisplatin for 24 h. The UM-UC-3 cells were treated with 1.47 μg/ml, while the T24 cells were treated with 1.82 μg/ml. Relative caspase 3/7 activity is indicated as percentage of untreated parental cells. The results are presented as the means ± SD of values obtained in three independent experiments. Statistical significance was calculated using the Student's t-tests when only two groups were compared or ANOVA tests when more than two groups were compared. *p < 0.05, **p < 0.01.

Chemoresistance is an important characteristic of CSCs^15^. Therefore, we investigated the role of WDR5 in apoptosis and chemoresistance by MTT assay and flow cytometry. As shown in Figure 5A, the cells transfected with WDR5 siRNA exhibited a lower resistance and IC_50_ to cisplatin than those transfected with control siRNA. In contrast, upregulation of WDR5 increased the resistance and IC_50_ value to cisplatin (Fig. 5B). Furthermore, we quantified apoptosis by staining cells with Annexin V and PI. WDR5 knockdown induced UM-UC3 and T24 cells apoptosis and significantly increased the percentage of apoptotic cells under cisplatin treatment (Fig. 5C–D, p < 0.05). The caspase 3/7 activity was upregulated in WDR5 knockdown cells and obviously increased when cells were treated with cisplatin (Fig. 5E). These results suggest that WDR5 may enhance bladder cancer cell anti-apoptosis and chemoresistance to cisplatin.

### WDR5 promotes tumorigenicity of bladder cancer cells *in vivo*

**Figure 6:**
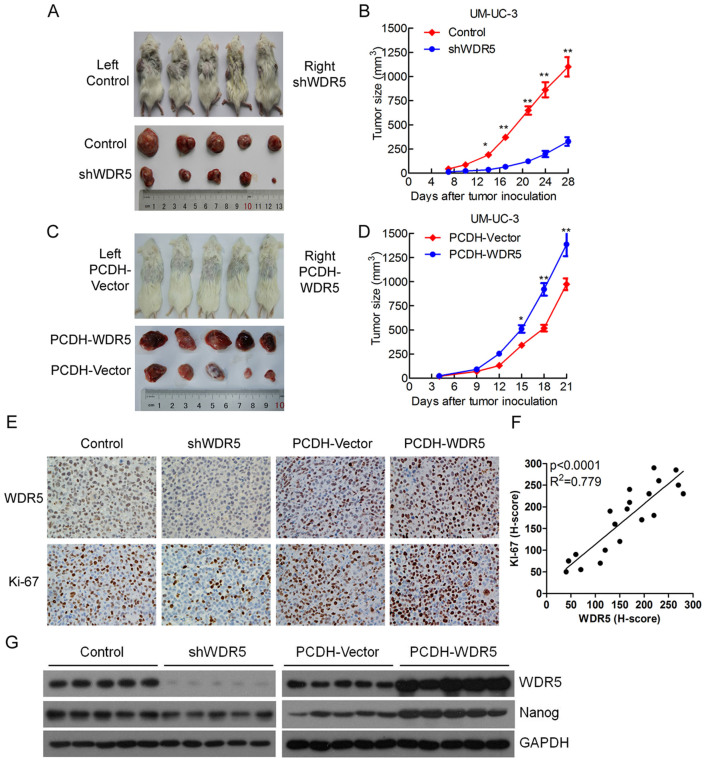
WDR5 promotes bladder tumor growth *in vivo*. (A and C) The images of animals and tumors are shown in this study. (B and D) The volume of tumor growth was measured every 3 days. The results are presented as the means ± SD of values (n = 5). Statistical significance was calculated using the Student's t-tests. *p < 0.05, **p < 0.01. (E) The expression of WDR5 and Ki67 in the tumor was examined by IHC. (F) The correlation between WDR5 protein level and Ki67 protein level was measured in the animal tumor tissues. The H-score values were subjected to Pearson correlation analysis (n = 20). (G) The expression of WDR5 and Nanog was detected in the xenograft tumors by Western blotting.

To further explore the effects of WDR5 in bladder cancer tumorigenesis *in vivo*, stable WDR5 knockdown, WDR5 overexpression or control UM-UC-3 cells were subcutaneously injected into NOD/SCID mice, and the tumor growth activity was measured. Interestingly, the growth of tumors derived from the WDR5 knockdown group was prominently suppressed compared with the control group from 2 weeks after tumor inoculation (Fig. 6A–B). In contrast, overexpression of WDR5 obviously accelerated the tumor growth *in vivo* compared with the PCDH-vector group (Fig. 6C and D). Moreover, the tumors derived from the WDR5 knockdown group exhibited lower expression of WDR5 and proliferation marker Ki67 than the control group. However, upregulation of WDR5 was associated with a higher Ki67 expression level in the PCDH-WDR5 group than the PCDH-Vector group (Fig. 6E). The WDR5 protein level was correlated with the Ki67 protein level in the animal tumor tissues (Fig. 6F). Furthermore, WDR5 knockdown decreased the expression of Nanog, whereas overexpression of WDR5 increased the expression of Nanog in the animal tumor tissues (Fig. 6G). These results indicate that WDR5 promots bladder cancer cell growth *in vivo* through the regulation of proliferation and self-renewal.

### The target genes of WDR5 were identified in bladder cancer

**Figure 7:**
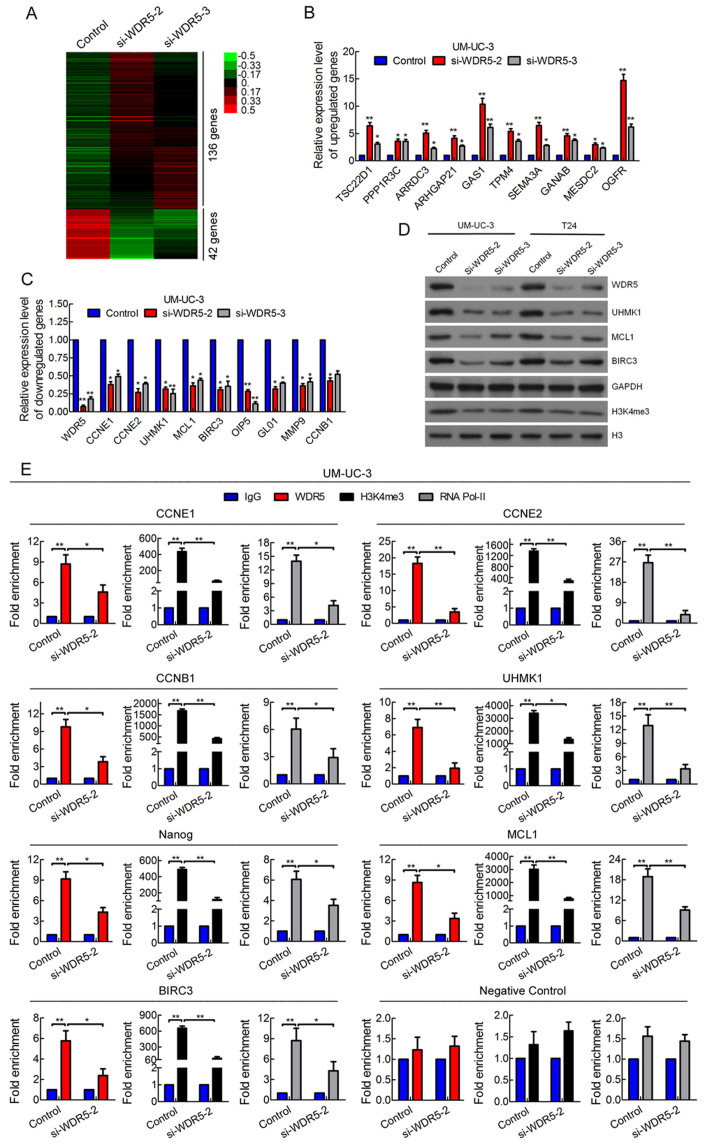
The target genes of WDR5 were identified in bladder cancer. (A) A heat map representing unsupervised hierarchical clustering of mRNA expression level in the UM-UC-3 cells transfected with control or WDR5 siRNA. Each column represents the indicated sample, and each row indicates one mRNA. Red and green colors indicate high and low expression, respectively. (B and C) The differentially expressed genes in the microarray were verified in UM-UC-3 cell by qRT-PCR. (D) The expression of candidate WDR5 target genes was detected by Western blotting. GAPDH and H3 were used as internal controls. The samples were derived from the same experiment and that blots were processed in parallel. The full-length blots are presented in Supplementary Figure 4. (E) ChIP analysis of IgG, WDR5, H3K4me3 and RNA polymerase-II status of candidate WDR5 target genes after knockdown assay. The results are presented as the means ± SD of values obtained in three independent experiments. Statistical significance was calculated using the Student's t-tests when only two groups were compared or ANOVA when more than two groups were compared. *p < 0.05, **p < 0.01.

To investigate the target genes of WDR5 in bladder cancer, we knocked-down WDR5 by si-WDR5-2 and si-WDR5-3 separately and monitored the changes in mRNA levels by microarray. There were 136 upregulated genes and 42 downregulated genes in both siRNAs of WDR5 compared with control siRNA (Fig. 7A, Tables S1). Next, we validated the expression of these genes in UM-UC-3 and T24 cells transfected with control or WDR5 siRNA by RT-qPCR. Consistent with above, some putative WDR5 target genes were obviously upregulated (Fig. 7B and Fig. S4A), whereas other genes were downregulated in WDR5 silenced cells (Fig. 7C and Fig. S4B). Moreover, the protein expression of MCL1, BIRC3 and UHMK1 was suppressed in WDR5 silenced cells (Fig. 7D). Previous reports have revealed that WDR5 regulates target gene activation mainly by mediating H3K4me3^9^,^10^. We also found that WDR5 knockdown obviously decreased the expression of H3K4me3, suggesting that WDR5 might regulate target genes in bladder cancer by H3K4me3 (Fig. 7D). Thus, we focused on the genes downregulated in WDR5 knockdown cells and a ChIP assay was performed to demonstrate H3K3me3 levels. As determined by ChIP-qPCR, WDR5 knockdown resulted in decreased H3K4me3 levels and location of WDR5 and RNA polymerase-II levels in the promoter regions of cyclin B1, cyclin E1, cyclin E2, UHMK1, MCL1, BIRC3 and Nanog, but not negative control, suggesting downregulation of these genes were directly regulated by WDR5 (Fig. 7E and Fig. S5). Taken together, these data indicate that WDR5 regulates target genes in bladder cancer by mediating H3K4me3 levels.

## Discussion

In recent years, it has become increasingly evident that aberrant proteins responsible for histone modifications play key roles in carcinogenesis. For example, EZH2 is identified as an oncogene in bladder cancer and lung cancer^16^,^17^, and Bmi1 regulates self-renewal of bladder cancer stem cells^18^. WDR5, a core component of the MLL/SET1 complexes, plays a critical role in embryonic stem cell self-renewal^11^, undifferentiated progenitor cells of the baboon subventricular zone^19^ and Epithelial-Mesenchymal Transition^12^. A recent study finds that H2A.Z is overexpressed in bladder cancer and activates oncogenic transcription by recruiting WDR5 and BPTF to its target genes^20^, suggesting that WDR5 may play a role in bladder cancer, but its expression pattern, role and mechanism in bladder cancer remain unclear. Here, we found that WDR5 was upregulated in bladder cancer tissues compared with normal tissues by IHC, and correlated with advanced tumor stage and overall survival of bladder cancer patients. Supporting our findings, a recent study found that WDR5 is overexpressed in prostate cancer tissue compared with normal tissues^13^. Taken together, high expression levels of WDR5 may serve as a novel molecular marker for bladder cancer. In addition, there are many questions to be elucidated, such as the correlation between WDR5 with metastasis and disease specify survival of bladder cancer patient. Answers to these questions will establish whether WDR5 could be used as an independent prognostic marker and criteria for patient selection.

As reported previously, WDR5 silencing reduces cell growth in breast cancer and prostate cancer^13^,^14^, but the detailed mechanism and role *in vivo* is still unknown. Through gain or loss of function, we discovered that WDR5 promoted bladder cancer cell proliferation *in vitro* and tumor growth *in vivo*, and that silencing WDR5 mainly induces the G0/G1 phase cell cycle arrest. The cell cycle is regulated by cyclins and cyclin-dependent kinases^21^. Cyclin E1 and cyclin E2 regulate the G1 to S-phase transition, while cyclin B1 regulates the G2 to M-phase transition^21^. Moreover, cyclin E is associated with high-grade, high-stage and invasive bladder cancer^22^,^23^. UHMK1 (also named KIS) is overexpressed in leukemia and promotes the G1 to S-phase transition^24^. Mechanistically, WDR5 knockdown obviously inhibited cyclin E1, cyclin E2 and UHMK1 leading to G0/G1 phase cell cycle arrest, which might disturb the effect of cyclin B1 downregulation on G2 to M-phase transition. However, another study showed that knockdown of MLL1, another core component of the MLL/SET1 complexes, suppressed HeLa cell proliferation by reducing the expression of cyclin B and inducing the G2/M phase cell cycle arrest^25^. These data suggest that WDR5 promotes bladder cancer cell proliferation *in vitro* and *in vivo* by regulating the cell cycle, but the role and mechanism are not the same as MLL1.

CSCs, a small subpopulation of cells in a tumor, can self-renew and differentiate into multiple lineages, and they possess strong tumor-initiating capacity. Therefore, CSCs are thought to be responsible for tumor initiation, progression, therapy resistance, relapse and metastasis^15^. CSCs have been widely identified in a number of malignancies, and the existence of CSCs in bladder cancer was found by Chan et al.^26^. Several studies have found that sphere culture is an effective way to enrich cancer stem cells^27^,^28^. In this study, we observed that WDR5 and pluripotency transcription factors were upregulated in UM-UC-3 and T24 spheres. Through gain or loss of function, we demonstrated that WDR5 promoted UM-UC-3 and T24 cells self-renewal *in vitro* and upregulated Nanog. Emerging evidence shows that Nanog is overexpressed in poorly differentiated tumors and correlated with poor survival outcome of patients with various types of cancer, including bladder cancer^29^,^30^. Moreover, Nanog plays a key role in CSCs self-renewal and targeting Nanog has shown the promising therapeutic potential in several types of cancer^31^,^32^. In our study, we identify that WDR5 directly activates Nanog by mediating its promoter H3K4me3 level. Taken together, our results suggest that WDR5 plays a vital role in self-renewal of bladder cancer cells by regulating Nanog.

Chemoresistance is an important characteristic of CSCs^15^. We found that WDR5 silencing increased cell apoptosis and decreased bladder cancer cells resistance to cisplatin. Conversely, overexpression of WDR5 enhanced chemoresistance to cisplatin. Moreover, we identified that WDR5 directly regulates important inhibitors of apoptotic proteins, MCL1^33^,^34^ and BIRC3^35^, by H3K4me3. The present study discovers that upregulation of MCL1 in cancers leads to chemoresistance, whereas targeting MCL1 is a novel strategy to overcome drug resistance in human^36^,^37^,^38^. Similarly, some researches find that BIRC3 silencing enhances chemotherapy sensitivity in several cancers^39^,^40^,^41^. A recent study reveals that a new compound named MM-401 blocks the MLL1-WDR5 interaction and shows a strong anticancer potential to mixed-lineage leukemia^42^. In sum, these findings indicate that WDR5 enhances bladder cancer cell anti-apoptosis and chemoresistance to cisplatin by regulating MCL1 and BIRC3, and it may be a potential target for drug development.

Given that WDR5 regulates various genes in bladder cancer, we identified the target genes of WDR5 by microarray, qPCR and Western blotting. The genes identified to be regulated by WDR5 were involved in a variety of biological functions, especially in cell cycle, self-renewal and anti-apoptosis. Moreover, we demonstrated that WDR5 regulated the cell cycle mainly by directly activating the transcription of cyclin E1, cyclin E2, cyclin B1 and UHMK1, whereas WDR5 promoted self-renewal by activating Nanog, WDR5 enhanced chemoresistance via mediating MCL1 and BIRC3 in bladder cancer cells by H3K4me3. Furthermore, we identified some target genes of WDR5, and further investigation is underway to elucidate how WDR5 regulates target genes contributing to cancer development.

In summary, it is our novel discovery that WDR5 is upregulated in bladder cancer, and promotes bladder cancer cell proliferation, self-renewal and chemoresistance via activating a series of oncogenes by H3K4me3. Therefore, WDR5 is a potential biomarker for bladder cancer and a promising target for drug development.

## Methods

### Tissue samples

Two tissue microarrays were purchased from Shanghai Outdo Biotech (Shanghai, China). One tissue microarray contains 31 bladder cancer specimens and 31 normal tissues. The other tissue microarray contains 59 bladder cancer specimens and 46 normal tissues. Follow-up data of these 59 cases were used for survival analysis. A total of 44 paraffin-embedded bladder cancer specimens were obtained from patients undergoing radical cystectomy at Sun Yat-sen Memorial Hospital between May 2012 and June 2013. All samples were evaluated and subjected to histological diagnosis by expert pathologists. All samples were collected with informed consent according to the internal review and ethics boards of the Sun Yat-sen University Cancer Center. Patient and tumor demographics are listed in Supplemental Table 2.

### IHC staining and scoring analyses

This experiment was conducted as previously described^43^. Briefly, paraffin sections were first deparaffinized and hydrated. For all antibodies, microwave antigen retrieval in citrate buffer (pH 6.0) was performed, and endogenous peroxidase activity was blocked by incubating the slides in 0.3% H_2_O_2_. After serial incubation with primary antibodies and secondary antibody, sections were developed with peroxidase and 3,3′-diaminobenzidine tetrahydrochloride. Sections were then counterstained with hematoxylin and mounted in nonaqueous mounting medium. The anti-WDR5 antibody (1:200, Abcam, ab176588) was used to detect WDR5 expression in bladder cancer tissues and normal tissues. The anti-WDR5 and anti-Ki67 antibodies (1:1000, Zhongshan Bio-Tech Co. Ltd, Beijing, China) were used to detect the expression of WDR5 and Ki67 in mice tumors. A negative control was performed by replacing the primary antibody with nonimmune IgG (DAKO). The human prostate cancer tissues were used as positive controls to test WDR5 antibody for IHC staining as shown in Supplemental Figure 1.

The expression of WDR5 in bladder specimens was blindly quantified by two pathologists using the histochemical score (H-score) as previously described^44^. Briefly, the intensity of staining (0 = negative, 1 = weak, 2 = moderate and 3 = strong) was multiplied by the percentage of positive cells (0%–100%), and the average H-score (0–300) of each tissue was obtained for statistical analysis. The samples were classed as low (H-score <200) or high (H-score ≥200) WDR5 expression. Images were visualized using a Nikon ECLIPSE Ti (Japan) microscope system and processed with Nikon software.

### Cell culture

The cell lines used in this study included the human bladder cancer cells UM-UC-3 and T24, and SV40-transformed kidney cell line 293T (ATCC, Manassas, VA). T24 cells were cultured in RPMI 1640 (Gibco, Shanghai, China), whereas UM-UC-3 and 293T cells were cultured in DMEM (Gibco, Shanghai, China). All media were supplemented with 10% FBS (Shanghai ExCell Biology, China) and 1% penicillin/streptomycin. Cells were grown in a humidified atmosphere of 5% CO_2_ at 37°C.

### Sphere culture

Sphere culture was conducted as previously described^45^. Briefly, cells were grown to 90% confluence, trypsinized, and plated at a density of 1,000 cells/ml in serum-free DMEM/F12 medium (Gibco, Shanghai, China) containing 20 ng/ml epidermal growth factor (EGF, R and D Systems, MN), 5 μg/ml insulin, 0.4% bovine serum albumin (Sigma, St. Louis, MO), and 2% B27 (Life Technologies) in ultra-low adhesion 12-well plates. After 10 days culture, the number of spheres was quantified by counting using 40× magnification under a phase contrast microscope (Olympus), and the cell number per sphere was quantified after dissociation to single cells. To propagate spheres *in vitro*, spheres were collected by gentle centrifugation, dissociated to single cells, and cultured to produce the next generation of spheres.

### RNA interference

SiRNA oligos that targeted WDR5 (1-GCUGGGAAUAUCCGAUGUATT, 2-GCUCAGAGGAUAACCUUGUTT, 3-CCCAGUCCAACCUUAUUGUTT) were purchased from GenePharma (Shanghai, China). SiRNA transfections were performed with 75 nM siRNA and Lipofectamine RNAimax (Life Technologies) following the manufacturer's instructions.

### Stable WDR5 overexpression or knockdown cell lines

The coding sequence of WDR5 was amplified by PCR and cloned in the pCDH-CMV-MCS-RFP-Puro vector (SBI). The pLKO.1 TRC cloning vector (Addgene plamid: 10878) was used to generate shRNA against WDR5 (GCTCAGAGGATAACCTTGTTT) or negative control (CCTAAGGTTAAGTCGCCCTCG). The lentivirus production and infection was conducted according to the manufacturer's protocol. As the vector used to overexpress WDR5 also expresses red fluorescent protein, the flow cytometry and EdU assay are not suitable for these transfected cells.

### Cell proliferation assay

The methyl thiazolyl tetrazolium (MTT; MTS, Promega) colorimetric assay was used to screen for cell viability. The cells transfected with control, WDR5 siRNA or the stable cells were seeded in 96-well plates at a density of 2 × 10^3^ cells/well. Then, the absorbance was measured at a wavelength of 490 nm for 5 days by SpectraMax M5 (Molecular Devices).

For the colony formation assay, the cells were seeded in a 6-well plate at a density of 1,000 cells per well after siRNA transfection, whereas the stable transfected cells were plated at a density of 500 cells per well. Approximately 10 days later, the clones were washed with 1 × PBS and stained with crystal violet for approximately 20 min. Finally, the clones were imaged and quantified.

For the cell cycle analysis, 48 h after transfection, cells were harvested and fixed in 70% ice cold ethanol and followed by RNase A treatment, stained with 50 μg/ml of propidium iodide for DNA content analysis in a FACSCaliber BD flow cytometer. The data were collected and processed using the BD FACSuite analysis software.

The EdU assay was performed according to the manufacturer's instructions (RiboBio, Guangzhou, China). Cells were seeded at 3 × 10^4^ cells/well in a 24-well plate and then transfected with control or WDR5 siRNA. Finally, 50 μM EdU was added to the plate, the cells were incubated for 2 h and the nuclei were stained with DAPI. The images were captured using an Olympus laser scanning microscope system.

### Chemosensitivity assay

For the chemosensitivity assay, transfected UM-UC-3 and T24 cells were treated with a series of different concentrations (0, 0.5, 1, 1.5, 2 and 2.5 μg/ml) of cisplatin (Sigma) for 48 h. The value was measured using the same method as MTS. For calculation of half inhibition concentration (IC_50_), data were fitted in Graph Pad Prism 5 (Graph Pad Software Inc., San Diego, CA, USA, 2005) and dose-response curve was plotted using the equation log(inhibitor) vs. response- Variable slope. It is also called a four-parameter dose-response curve: 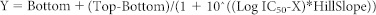
46,47,48.

### Apoptosis analysis

The cells transfected with control or WDR5 siRNA 24 h later were treated with 0 or 2 μg/ml cisplatin for 24 h. The cells were then collected, washed with PBS and the cell apoptosis was analyzed with Annexin V-FITC and PI (KeyGEN Biotech, Nanjing, China) staining in a FACSCaliber BD flow cytometer.

### Detection of caspase-3/7 activity

Enzymatic activity of caspase-3/7 was measured using the Caspase-Glo 3/7 Assay kit (Promega, Shanghai, China) according to the manufacturer's instruction. Briefly, the cells transfected with control or WDR5 siRNA were seeded in 96-well plates and treated with or without IC_50_ concentration of parental cells cisplatin for 24 h. The UM-UC-3 cells were treated with 1.47 μg/ml, while the T24 cells were treated with 1.82 μg/ml. Next, the cells were lysed and incubated with 100 μL of Apo-ONE Caspase-3/7 reagent (substrate and buffer in the ratio of 1:100). After 1 h incubation in the dark at room temperature, the fluorescence of each well was measured at 485–520 nm by reading in SpectraMax M5 (Molecular Devices). The experiments were replicated three times.

### RNA isolation and quantitative RT-PCR

Total RNA was extracted from cells using Trizol reagent (Invitrogen) according to the manufacturer's protocol. Total RNA was used for reverse transcription with the PrimerScript RT-PCR kit (TaKaRa Biotechnology, Dalian, China). Quantitative RT-PCR was conducted using a standard SYBR Green PCR kit (Roche) protocol with a LightCycler 480 real-time instrument (Roche). The relative expression was calculated using the 2^−ΔΔCt^ method. The transcription level of GAPDH was used as an internal control. All specific primers are listed in Supplementary Table 3.

### Western blotting

The total protein extracted from the samples was resolved on 10% sodium dodecyl sulfate–polyacrylamide gels and electrophoretically transferred to a polyvinylidene fluoride membrane. Blots were blocked with 5% skim milk followed by incubation with antibodies. Primary antibodies specific to WDR5, H3K4me3, Oct4, Sox2 and Nanog (1:1000, Abcam), cyclin A, cyclin B1, cyclin D1, cyclin E1, cyclin E2, cyclin H, H3, MCL1 and GAPDH (1:1000, CST), UHMK1 and BIRC3 (1:1000, ABGENT) were used. The blots were then incubated with goat anti-rabbit or anti-mouse secondary antibody (CST) and visualized using enhanced chemiluminescence.

### Tumorigenesis study

NOD/SCID mice (5 weeks of age, 18–20 g, Vital River Laboratories, Beijing, China) were housed in sterile filter-capped cages. A total of 3 × 10^6^ cells were injected subcutaneously on the right or left side of the dorsum and five mice were used. Twenty-one and twenty-eight days post-implantation, the mice were euthanized and tumors were surgically dissected, respectively. The tumor specimens were fixed in 4% paraformaldehyde.

### Microarray analysis

The PrimeView™ Human Gene Expression Array (Affymetrix) was used in this study and performed by CapitalBio Corporation (Beijing, China) according to the manufacturer's instructions. The arrays were scanned on a GeneChip Scanner 3000, and the data were analyzed using GeneChip Operating software (GCOS 1.4). All primary data in microarray analysis are available at the Gene Expression Omnibus (GEO accession: GSE59132).

### Chromatin immunoprecipitation (ChIP)

Cells were transfected with si-WDR5-2 or control siRNA for 72 h. Chromatin immunoprecipitation was conducted with the EZ-Magna ChIP A/G kit (Millipore) according to the manufacturer's instructions. Briefly, 1 × 10^6^ cells were used for each reaction. Cells were fixed in 1% formaldehyde at room temperature for 10 min, the nucleus was isolated with nuclear lysis buffer (Millipore) supplemented with protease inhibitor cocktail (Millipore). Chromatin DNA was sonicated and sheared to a length between 200 bp and 1000 bp. The sheared chromatin was immunoprecipitated at 4°C overnight with anti-WDR5 (Abcam, ab56919) and anti-H3K4me3 (Abcam, ab8580) antibodies. Normal rabbit IgG was used as a negative control and the anti-RNA pol-II (Millipore) antibody was used as a positive control. The WDR5, H3K4me3 and RNA pol-II protein level in the ChIP assays were detected by Western blotting (Fig S6). The negative control primers were used to detect the DNA fragment out of genes as a distal region control. Primers for ChIP-qPCR are listed in Supplementary Table 3.

### Statistical analyses

Data were presented as the mean ± SD from three independent experiments. Two-tailed Student's t-tests and one-way analysis of variance (ANOVA) were used to evaluate the data. The differences between groups of WDR5 expression in bladder cancer tissues were analyzed using the Chi-squared test (χ^2^ test). The log-rank test was used to explore the associations between WDR5 expression and the overall survival of bladder cancer patients. The correlation between WDR5 protein level and Ki67 protein level was analysed by Pearson correlation analysis. All of the statistical analyses were performed with SPSS 19.0. The difference was considered to be statistically significant at *p < 0.05 and **p < 0.01.

### Ethics statement

All of the animal care and experimental procedures were approved by the Institutional Animal Care and Use Committee of Sun Yat-sen University. The methods were carried out in accordance to the approved guidelines.

## Author Contributions

J.H. and T.L. conceived and designed the experiments. X.C., W.X. and P.G. participated in the experiment, performed data analysis and wrote the manuscript. W.D., G.Z. and W.H. collected clinical samples and information. Q.C., B.W. and Y.X. performed IHC and clinical and pathological analyses.

## Supplementary Material

Supplementary Information Supplement

## Figures and Tables

**Table 1:** Association between WDR5 expression and clinicopathological features of bladder caner

		WDR5 expression in urinary bladder cancer tissues		
Variables	Patient No.(n)	Low	High	P value
**Gender**				0.418
Male	117	39	78	
Female	17	4	13	
**Age(Year)**				
≤65	69	19	50	0.245
>65	65	24	41	
**Tumor size(cm)**				0.689
≤3	50	15	35	
>3	84	28	56	
**Tumor stage**				0.005*
CIS,Ta,T1	38	19	19	
T2–4	96	24	72	
**Tumor grade**				
Grade 1,2	50	10	30	0.130
Grade 3	84	23	61	
**Lymphnodes status**				0.172
Negative	117	40	77	
Positive	17	3	14	

*P < 0.05 is considered significant.
