# Supplementary material for: Upregulated WDR5 promotes proliferation, self-renewal and chemoresistance in bladder cancer via mediating H3K4 trimethylation
Source: Sci Rep. 2015 Feb 6;5:8293. doi: 10.1038/srep08293 (PMC4319178; doi:10.1038/srep08293)

**Upregulated WDR5 promotes proliferation, self-renewal and chemoresistance in bladder cancer via mediating H3K4 trimethylation**

Xu Chen^1, 2,a^, Weibin Xie^1, 2,a^, Peng Gu^1, 2,a^, Qingqing Cai^3^, Bo Wang^1, 2^, Yun Xie^1^, Wen Dong^1^, Wang He^1^, Guangzheng Zhong^1^, Tianxin Lin^1,2,*^ & Jian Huang^1,*^

^1^ Department of Urology, Sun Yat-sen Memorial Hospital, Sun Yat-sen University, Guangzhou, China

^2^ Guangdong Provincial Key Laboratory of Malignant Tumor Epigenetics and Gene Regulation, Sun Yat-Sen Memorial Hospital, Sun Yat-Sen University,Guangzhou, China

^3^ Department of Internal Medicine, Sun Yat-sen University Cancer Center, Guangzhou, China

^a^ These three authors contributed equally to this work.

Corresponding authors Address: Department of Urology, Sun Yat-sen Memorial Hospital, 107th Yanjiangxi Road, Guangzhou, China. Jian Huang, Tel: 86-13600054833, fax: 86-20-81332336. E-mail: [urolhj@sina.com](mailto:urolhj@sina.com) and Tianxin Lin, Tel: 86-13724008338, fax: 86-20-81332336. E-mail: [tianxinl@sina.com](mailto:tianxinl@sina.com).

Supplementary information

**Methods**

**Nanog was overexpressed in bladder cancer cells.** The coding sequence of Nanog was amplified by PCR and cloned in the pcDNA3.0 (Invitrogen, Life Technologies). Transfections were performed with 1.5 μg pcDNA3.0 or pcDNA3.0-Nanog and X- tremeGENE (Roche) in 6-well plates following the manufacturer’s instructions. After 24 hours, the transfected cells were performed MTT, colony and sphere formation assay as previously described.

**Figure legends**

**Supplemental Figure 1. (A)** The human prostate cancer tissues were used as positive controls to test WDR5 antibody for IHC staining. (B) Negative control samples included replacement of the primary antibody with nonimmune IgG in bladder normal urothelium and cancer tissue by IHC. Representative samples are shown at 400× magnification.

**Supplemental Figure 2.** Efficiency of WDR5 stable knockdown in UM-UC-3 and T24 cells by lentivirus was verified by Western blotting.

**Supplemental Figure 3. (A)** Efficiency of Nanog overexpression in UM-UC-3 and T24 cells by transfections was verified by Western blotting. (B) The self-renewal capacity of UM-UC-3 and T24 cells after transfection of Nanog or control vector was measured by spheres formation. (C and D) The number of spheres reflected the quantity of cells capable of *in vitro* self-renewal, whereas the number of cells/sphere measured the self-renewal capacity of each sphere-generating cell. The results are presented as the means ± SD of values obtained in three independent experiments. Statistical significance was calculated using the Student’s t-tests. *p < 0.05, **p < 0.01.

**Supplemental Figure 4.** The upregulated genes (A) or downregulated genes (B) in the microarray were verified in T24 cell by qRT-PCR. The results are presented as the means ± SD of values obtained in three independent experiments. Statistical significance was calculated using the ANOVA. *p < 0.05, **p < 0.01.

**Supplemental Figure 5.** ChIP analysis of IgG, WDR5, H3K4me3 and RNA polymerase-II status of candidate WDR5 target genes after knockdown assay. The results are presented as the means ± SD of values obtained in three independent experiments. Statistical significance was calculated using the Student’s t-tests. *p < 0.05, **p < 0.01.

**Supplemental Figure 6.** The WDR5, H3K4me3 and RNA pol-II protein level in the ChIP assays were detected by Western blotting.

**Supplement Table 1.** Differentially expressed mRNAs in microarray.

**Supplemental Table 2.** Characteristics of patients and tumors in tissue specimens

**Supplemental Table 3.** List of the primer sequences for polymerasechain reaction studies.

**Supplemental Figure 4.** The full-length blots of manuscript are presented.

**Supplemental Figure 1.**

**
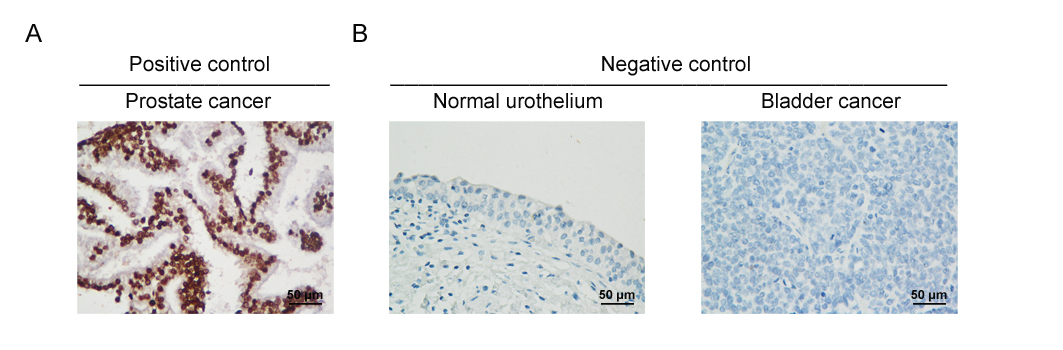
**

**Supplemental Figure 2.**


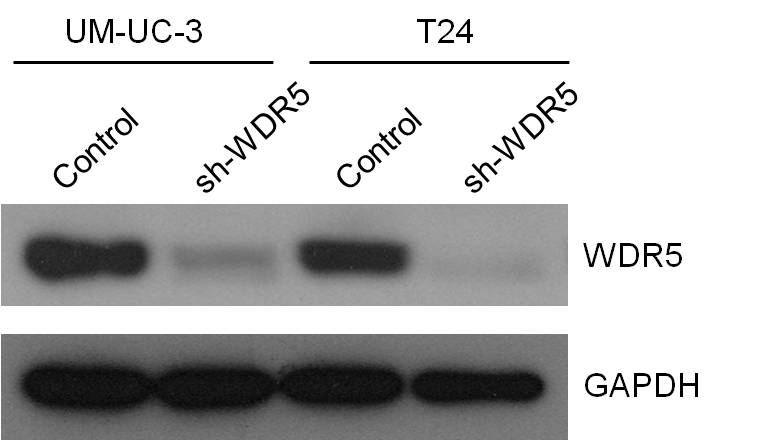


**Supplemental Figure 3.**

**
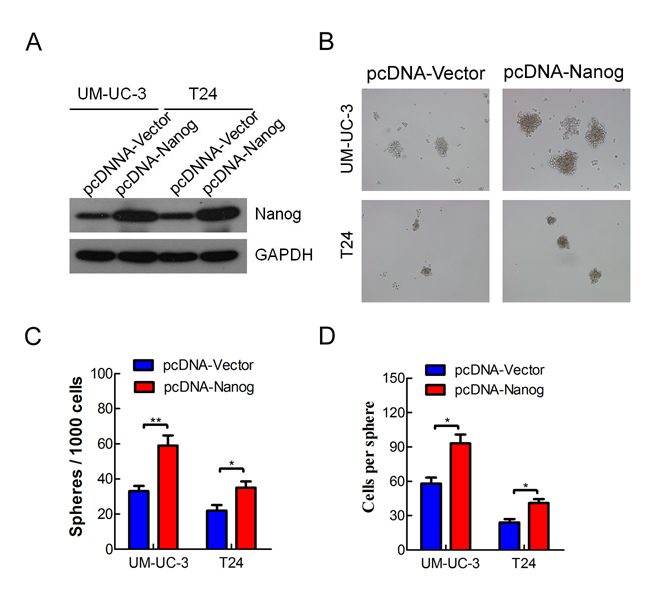
**

**Supplemental Figure 4.**


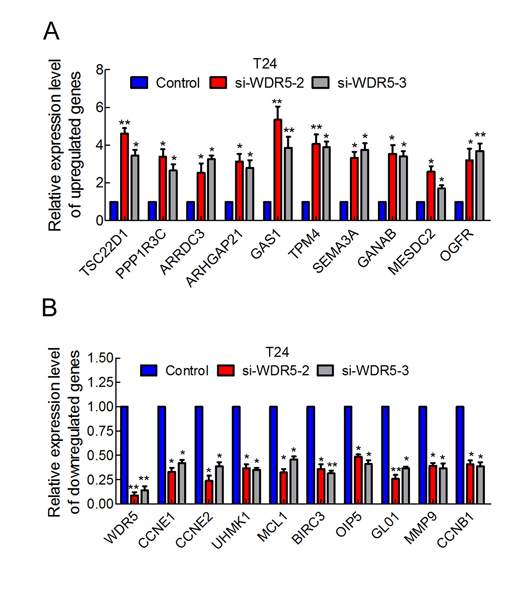


**Supplemental Figure 5.**

**
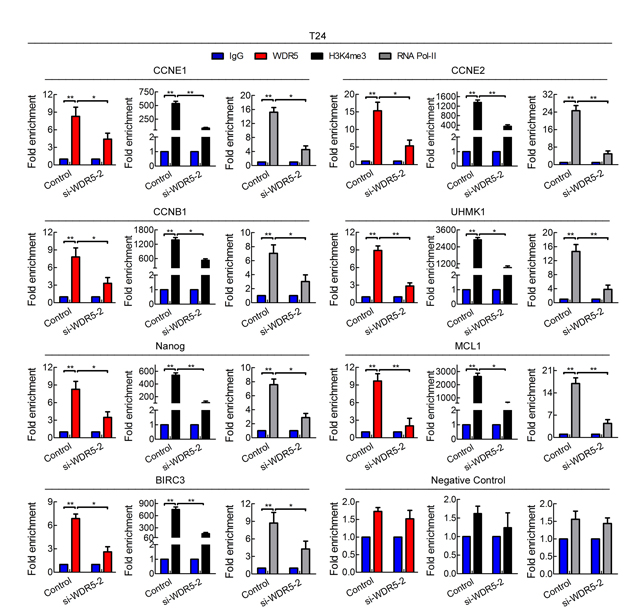
**

**Supplemental Figure 6.**

**
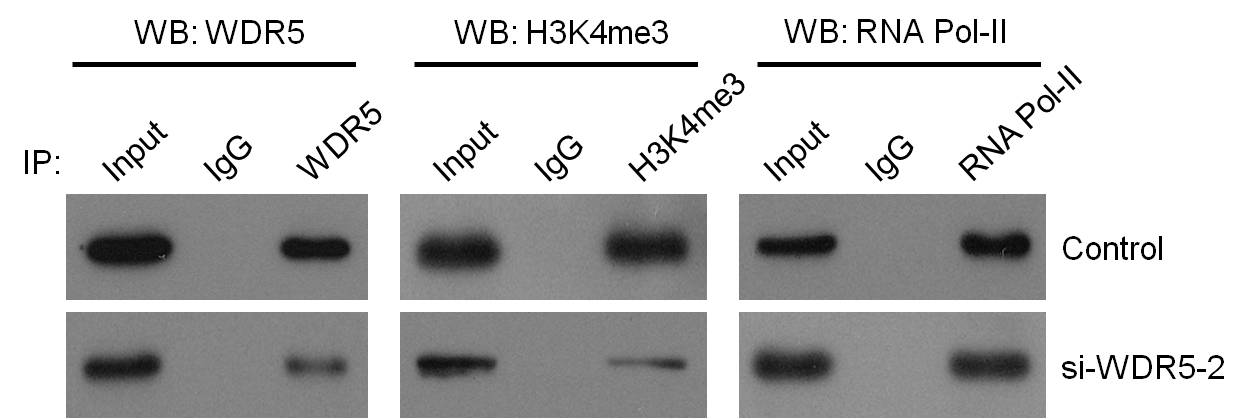
**

**Supplement Table 1. Differentially expressed mRNAs in microarray.**

| Gene name | si-WDR5-2 vs control Ratio | si-WDR5-3 vs control Ratio | up/down |
| --- | --- | --- | --- |
| | CBS | | --- | | CBS | | CBS | | OGFR | | HIST1H2BD | | C7orf55 /// LUC7L2 | | CITED2 | | TPM4 | | TPM4 | | ZNF664 | | TPM4 | | SPANXC | | DDIT4 | | HIST1H2BD | | ZNF664 | | HIST1H2BD | | SEMA3A | | SPANXA1 | | SEMA3A | | TSC22D1 | | TUFT1 | | ZNF664 | | TRAK1 | | HIST1H2BK | | COL4A6 | | ANXA4 | | ARRDC3 | | CCDC85B | | ANXA4 | | ANXA4 | | EFNB2 | | POLD4 | | ARRDC3 | | POLD4 | | CD24 | | POLD4 | | C15orf63 /// SERF2 | | TMEM93 | | RAD23A | | KIF1C | | TCF21 | | PRR16 | | RAD23A | | CASP7 | | MESDC2 | | MARCKS | | FN1 | | CFH /// CFHR1 | | MKNK2 | | ODZ3 | | COL4A5 | | C6orf120 | | TXNIP | | RAD23A | | CLIP1 | | ZNF227 | | CCDC53 | | SOX12 | | TBC1D9 | | MALAT1 | | WBP4 | | CFH | | RAD23A | | RGS19 | | NEXN | | ANXA4 | | IGFBP3 | | RPL26L1 | | LONP1 | | PCDH10 | | NOLC1 | | ANKRD11 | | ARHGAP21 | | GANAB | | DCI | | ANXA4 | | LONP1 | | DENND5A | | TCF3 | | SPANXB1 | | SOX12 | | RRBP1 | | ISCA1 | | COL4A1 | | HAS2 | | MED11 | | LPCAT1 | | ERO1L | | CFH /// CFHR1 | | LONP1 | | ANKRD11 | | CUL4B | | JARID2 | | NEXN | | STYXL1 | | POSTN | | STYXL1 | | NPAS3 | | TRPS1 | | TXNIP | | SPANXB1 | | TOMM22 | | PNRC1 | | DUSP16 | | NDUFS2 | | KITLG | | SPANXB1 | | NDUFS2 | | STYXL1 | | GANAB | | ISCA1 | | KHNYN | | GAS1 | | POLR3GL | | TUBA1A | | PTPRK | | MARCKS | | AGA | | PPP1R3C | | PPP1R14B | | FAM127A | | FAM119A | | PTPRK | | NEXN | | SLC17A5 | | GOLGA2 | | C6orf47 | | TUBA1A | | TPM4 | | ARL4C | | SNORA12 | | POLR2D | | MAP4K4 | | NEXN | | ANXA4 | | TSPAN31 | | PPP1R9B | | ZKSCAN1 | | ANXA4 | | NOLC1 | | AHNAK2 | | KDM2B | | FAM3C | | CPA4 | | FAM3C | | R3HCC1 | | EXTL3 | | ZBED4 | | EPN1 | | PHACTR2 | | AHNAK2 | | TOMM22 | | RAC1 | | NOL12 /// TRIOBP | | XRCC4 | | ARL4C | | C9orf86 | | GANAB | | VOPP1 | | RAC1 | | TRIOBP | | AHNAK2 | | G3BP2 | | EXOC5 | | NDRG1 | | ZCCHC2 | | BICD2 | | CCDC92 | | AES | | C2CD2 | | COL5A1 | | COL5A1 | | BICD2 | | REEP5 | | AKT1 | | MEX3D | | TSHZ1 | | ICMT | | ZDHHC16 | | PHACTR2 | | BRD3 | | CTSB | | PHACTR2 | | MSRB3 | | BICD2 | | CDK14 | | ZDHHC16 | | SCARNA14 | | PDE8A | | NFKBIA | | NFKBIA | | TMEM22 | | TMEM22 | | ATP6V1C1 | | SERBP1 | | CDKN1A | | SH2B3 | | CDKN1A | | LMAN1 | | RRM2 | | CCT7 | | CD82 | | UHMK1 | | CD82 | | FAM91A1 /// FAM91A2 | | PLEK2 | | LMAN1 | | ZNF697 | | MCL1 | | RPS15A | | GLO1 | | RPL37 | | DLEU2 | | MCL1 | | RPIA | | NEK7 | | CCNE1 | | PLAU | | DLEU2 /// DLEU2L | | CMPK1 | | RPIA | | SERBP1 | | BAG4 | | RPS14 | | H2AFV | | RPL7L1 | | CMPK1 | | METAP2 | | CCNE2 | | RPL7L1 | | RPL7L1 | | RPL7L1 | | TMCC3 | | MNS1 | | RPL7L1 | | RPS14 | | GLO1 | | EHD1 | | CA2 | | TMCC3 | | MMP9 | | RPL35A | | OIP5 | | GLO1 | | LOC401127 /// WDR5 | | NETO2 | | WDR5 | | WDR5 | | NCEH1 | | | 7.425 | | --- | | 7.0559 | | 4.2068 | | 3.9207 | | 3.7011 | | 3.5743 | | 3.529 | | 3.4995 | | 3.4846 | | 3.4052 | | 3.3927 | | 3.358 | | 3.3361 | | 3.332 | | 3.3096 | | 3.3038 | | 3.2096 | | 3.1945 | | 3.1785 | | 3.138 | | 3.134 | | 3.1152 | | 2.9128 | | 2.8699 | | 2.8365 | | 2.8259 | | 2.8073 | | 2.7822 | | 2.759 | | 2.7138 | | 2.69 | | 2.67 | | 2.6695 | | 2.6695 | | 2.6589 | | 2.6461 | | 2.6374 | | 2.6314 | | 2.6268 | | 2.6182 | | 2.6154 | | 2.5899 | | 2.5719 | | 2.5581 | | 2.5267 | | 2.526 | | 2.5128 | | 2.5048 | | 2.4995 | | 2.4795 | | 2.4608 | | 2.4492 | | 2.4377 | | 2.4357 | | 2.4342 | | 2.4316 | | 2.4151 | | 2.3846 | | 2.3803 | | 2.3741 | | 2.3695 | | 2.3687 | | 2.3593 | | 2.3554 | | 2.349 | | 2.3417 | | 2.3339 | | 2.3264 | | 2.3256 | | 2.3192 | | 2.3191 | | 2.3151 | | 2.3118 | | 2.3011 | | 2.2995 | | 2.2908 | | 2.2887 | | 2.288 | | 2.2839 | | 2.2801 | | 2.2785 | | 2.2745 | | 2.269 | | 2.2657 | | 2.263 | | 2.254 | | 2.2534 | | 2.2524 | | 2.2448 | | 2.2417 | | 2.241 | | 2.2351 | | 2.2269 | | 2.2239 | | 2.2227 | | 2.2176 | | 2.2131 | | 2.21 | | 2.2085 | | 2.2043 | | 2.1923 | | 2.1855 | | 2.183 | | 2.177 | | 2.176 | | 2.1735 | | 2.165 | | 2.1648 | | 2.1615 | | 2.1577 | | 2.1539 | | 2.1502 | | 2.1475 | | 2.1407 | | 2.1236 | | 2.1188 | | 2.1176 | | 2.1158 | | 2.1135 | | 2.1096 | | 2.1015 | | 2.0989 | | 2.0973 | | 2.0919 | | 2.0908 | | 2.0884 | | 2.0656 | | 2.0635 | | 2.0539 | | 2.0502 | | 2.0427 | | 2.0418 | | 2.0403 | | 2.0393 | | 2.0306 | | 2.0107 | | 2.0105 | | 2.0064 | | 2.0005 | | 2 | | 1.9984 | | 1.994 | | 1.9758 | | 1.9662 | | 1.9581 | | 1.9495 | | 1.9434 | | 1.9429 | | 1.9371 | | 1.9361 | | 1.9358 | | 1.9278 | | 1.9252 | | 1.9226 | | 1.9173 | | 1.9157 | | 1.9134 | | 1.8925 | | 1.8707 | | 1.8685 | | 1.8657 | | 1.8359 | | 1.8249 | | 1.8172 | | 1.8152 | | 1.8022 | | 1.7906 | | 1.7847 | | 1.7622 | | 1.7619 | | 1.7476 | | 1.7364 | | 1.7247 | | 1.7196 | | 1.7146 | | 1.7078 | | 1.7002 | | 1.6742 | | 1.6592 | | 1.6587 | | 1.6416 | | 1.6326 | | 1.6036 | | 1.6034 | | 1.5863 | | 1.5735 | | 1.5592 | | 1.5321 | | 1.5014 | | 0.6663 | | 0.6647 | | 0.6404 | | 0.637 | | 0.6346 | | 0.6327 | | 0.5883 | | 0.5866 | | 0.5798 | | 0.5333 | | 0.5111 | | 0.5056 | | 0.4997 | | 0.4982 | | 0.4949 | | 0.4939 | | 0.4932 | | 0.4915 | | 0.483 | | 0.4822 | | 0.4771 | | 0.4707 | | 0.4671 | | 0.4643 | | 0.4641 | | 0.4619 | | 0.4618 | | 0.4582 | | 0.4576 | | 0.4492 | | 0.4478 | | 0.4388 | | 0.4337 | | 0.4333 | | 0.4267 | | 0.4227 | | 0.4198 | | 0.4155 | | 0.4096 | | 0.4089 | | 0.4036 | | 0.3989 | | 0.3968 | | 0.3952 | | 0.391 | | 0.3882 | | 0.3783 | | 0.3715 | | 0.3624 | | 0.3454 | | 0.3418 | | 0.3403 | | 0.3372 | | 0.3286 | | 0.3279 | | 0.277 | | 0.2523 | | 0.2307 | | 0.201 | | 0.1824 | | | 2.4458 | | --- | | 2.5221 | | 1.7269 | | 2.3873 | | 2.9632 | | 2.9538 | | 1.6717 | | 3.8763 | | 3.6688 | | 5.0983 | | 3.9904 | | 1.6616 | | 1.6852 | | 2.4794 | | 5.0771 | | 2.3026 | | 2.0934 | | 1.6548 | | 1.9949 | | 3.2555 | | 1.5378 | | 4.8515 | | 2.099 | | 1.5734 | | 1.9519 | | 1.912 | | 2.0145 | | 1.5232 | | 1.8614 | | 1.8124 | | 1.5279 | | 1.5704 | | 2.2556 | | 1.6535 | | 2.0889 | | 1.5883 | | 1.8966 | | 2.3954 | | 2.981 | | 3.377 | | 1.519 | | 1.5332 | | 2.6731 | | 1.6697 | | 2.3585 | | 1.8605 | | 1.931 | | 1.7418 | | 2.7414 | | 2.3588 | | 2.0812 | | 1.5192 | | 1.6965 | | 2.6266 | | 2.1109 | | 2.0504 | | 1.6329 | | 1.9177 | | 2.2183 | | 1.7033 | | 1.7364 | | 1.5955 | | 2.7131 | | 2.7914 | | 1.8073 | | 1.6913 | | 1.6783 | | 1.5532 | | 1.7659 | | 1.993 | | 1.817 | | 1.7562 | | 2.2928 | | 2.7871 | | 1.7775 | | 1.8487 | | 1.7432 | | 1.7432 | | 2.8537 | | 1.7739 | | 1.8849 | | 2.0147 | | 1.8515 | | 1.7209 | | 1.5838 | | 1.9269 | | 2.3308 | | 1.5412 | | 1.6325 | | 1.6935 | | 1.7135 | | 1.5874 | | 1.541 | | 1.6685 | | 1.6594 | | 1.8398 | | 1.7325 | | 1.7982 | | 1.7306 | | 1.5625 | | 1.7468 | | 2.5518 | | 1.8043 | | 1.5194 | | 2.0231 | | 1.5019 | | 1.7144 | | 2.0913 | | 1.5794 | | 2.4733 | | 1.9209 | | 1.8648 | | 2.3323 | | 1.8051 | | 2.2286 | | 1.8183 | | 1.8478 | | 1.5464 | | 3.2406 | | 2.2094 | | 1.5433 | | 1.8671 | | 1.8575 | | 1.7193 | | 1.6011 | | 1.627 | | 1.7836 | | 2.1327 | | 1.7888 | | 2.1551 | | 1.852 | | 1.6756 | | 1.6737 | | 1.6218 | | 1.7035 | | 1.5993 | | 1.8687 | | 1.7675 | | 1.6512 | | 1.6516 | | 2.2153 | | 2.4115 | | 2.488 | | 2.085 | | 2.3217 | | 2.2532 | | 2.389 | | 2.0512 | | 2.1416 | | 2.0258 | | 2.1767 | | 2.2954 | | 2.1884 | | 3.2951 | | 2.0495 | | 2.0704 | | 2.1545 | | 2.1956 | | 2.4276 | | 2.2049 | | 2.7998 | | 2.0551 | | 2.6303 | | 2.0116 | | 2.473 | | 2.1305 | | 3.1977 | | 2.1733 | | 2.0947 | | 2.0409 | | 2.2961 | | 2.0139 | | 2.9057 | | 2.9493 | | 2.0047 | | 2.2381 | | 2.1084 | | 2.6781 | | 2.1867 | | 2.4713 | | 2.0001 | | 2.5291 | | 2.2751 | | 2.4006 | | 2.6594 | | 4.2218 | | 2.0639 | | 2.0264 | | 2.0078 | | 0.4653 | | 0.4495 | | 0.4921 | | 0.4597 | | 0.4652 | | 0.4325 | | 0.424 | | 0.4821 | | 0.4187 | | 0.393 | | 0.3924 | | 0.4878 | | 0.5721 | | 0.4384 | | 0.5731 | | 0.517 | | 0.6387 | | 0.368 | | 0.5288 | | 0.5344 | | 0.5342 | | 0.6227 | | 0.5365 | | 0.6511 | | 0.456 | | 0.3312 | | 0.6699 | | 0.5281 | | 0.5281 | | 0.6112 | | 0.605 | | 0.3858 | | 0.6062 | | 0.662 | | 0.3512 | | 0.6179 | | 0.5832 | | 0.5025 | | 0.4807 | | 0.6191 | | 0.5535 | | 0.6032 | | 0.5894 | | 0.6296 | | 0.6597 | | 0.5863 | | 0.3276 | | 0.6588 | | 0.5186 | | 0.5181 | | 0.6644 | | 0.2463 | | 0.3506 | | 0.2609 | | 0.612 | | 0.2703 | | 0.5654 | | 0.3234 | | 0.3111 | | 0.5252 | | | up | | --- | | up | | up | | up | | up | | up | | up | | up | | up | | up | | up | | up | | up | | up | | up | | up | | up | | up | | up | | up | | up | | up | | up | | up | | up | | up | | up | | up | | up | | up | | up | | up | | up | | up | | up | | up | | up | | up | | up | | up | | up | | up | | up | | up | | up | | up | | up | | up | | up | | up | | up | | up | | up | | up | | up | | up | | up | | up | | up | | up | | up | | up | | up | | up | | up | | up | | up | | up | | up | | up | | up | | up | | up | | up | | up | | up | | up | | up | | up | | up | | up | | up | | up | | up | | up | | up | | up | | up | | up | | up | | up | | up | | up | | up | | up | | up | | up | | up | | up | | up | | up | | up | | up | | up | | up | | up | | up | | up | | up | | up | | up | | up | | up | | up | | up | | up | | up | | up | | up | | up | | up | | up | | up | | up | | up | | up | | up | | up | | up | | up | | up | | up | | up | | up | | up | | up | | up | | up | | up | | up | | up | | up | | up | | up | | up | | up | | up | | up | | up | | up | | up | | up | | up | | up | | up | | up | | up | | up | | up | | up | | up | | up | | up | | up | | up | | up | | up | | up | | up | | up | | up | | up | | up | | up | | up | | up | | up | | up | | up | | up | | up | | up | | up | | up | | up | | up | | up | | up | | up | | down | | down | | down | | down | | down | | down | | down | | down | | down | | down | | down | | down | | down | | down | | down | | down | | down | | down | | down | | down | | down | | down | | down | | down | | down | | down | | down | | down | | down | | down | | down | | down | | down | | down | | down | | down | | down | | down | | down | | down | | down | | down | | down | | down | | down | | down | | down | | down | | down | | down | | down | | down | | down | | down | | down | | down | | down | | down | | down | | down | |

**Supplemental Table 2.** Characteristics of patients and tumors in tissue specimens

|  | | Tumor tissues | | Tumor tissues for survival analysis |
| --- | --- | --- | --- | --- |
| **Patients(N)** | | 134 | | 59 |
| **Gender N(%)** | |  | |  |
| Male | | 117(87.3) | | 51(86.4) |
| Female | | 17(12.7) | | 8(13.6) |
| **Age(Year)** | |  | |  |
| Median(range) | | 66(31-85) | | 71(44-85) |
| Mean±SD | | 65.9±10.6 | | 68.5±10.0 |
| **Tumor size N(%)** | |  | |  |
| ≤3cm | | 50(37.3) | | 23(39.0) |
| >3cm | | 84(62.7) | | 36(61.0) |
|  |  | | | |
| **NMIBC** | | 38(28.4) | | 16(27.1) |
| pTa | | 6(4.5) | | 1(1.7) |
| pTis | | 7(5.2) | | 4(6.8) |
| pT1 | | 25(18.7) | | 1118.6() |
| **MIBC** | | 96(71.6) | | 43(72.0) |
| pT2 | | 41(30.6) | | 18(30.5) |
| pT3 | | 35(26.1) | | 21(35.6) |
| pT4 | | 20(14.9) | | 4(6.8) |
| **Pathologic tumor grade** **N (%)** |  | |  | |
| Low grade /G1+G2 | | 50(37.3) | | 20(33.9) |
| High grade/G3 | | 84(62.7) | | 39(66.1) |
| **Lymphnodes status N(%)** | |  | |  |
| Negative | | 117(87.3) | | 53(89.8) |
| Positive | | 17(12.7) | | 6(10.2) |

**Supplement Table 3.** List of the primer sequences for polymerasechain reaction studies.

| Target | Forward Sequence (5’-3’) | Reverse Sequence (5’-3’) |
| --- | --- | --- |
| **Primer for RT-qPCR**  WDR5 | AATATCCGATGTAGCCTGGTC | TTGGACTGGGGATTGAAGTTG |
| GAPDH  Oct4 | CAAGGCTGAGAACGGGAAG  TATGCAAAGCAGAAACCCTCGTG | TGAAGACGCCAGTGGACTC  CTGGCGCCGGTTACAGAACCA |
| Sox2 | ATGACCAGCTCGCAGACCTAC | TTGACCACCGAACCCATGGAG |
| Nanog | TCCAGCAGATGCAAGAACTCTCCA | CACACCATTGCTATTCTTCGGCCA |
| CCNA1 | CCAAGCAAGGGTTTGACATC | GTGTGCCGGTGTCTACTTCA |
| CCNB1 | TAAGGCGAAGATCAACATGG | TTACCAATGTCCCCAAGAGC |
| CCND1 | CAAATGTGTGCAGAAGGAGGT | GAAGCGGTCCAGGTAGTTCA |
| CCNE1 | CAGCCTTGGGACAATAATGC | TGCACGTTGAGTTTGGGTAA |
| CCNE2 | CGTTTACAAGCTAAGCAGCAG | CCTGGGTAGTTTTCCTCTTC |
| MCL1 | AAGGACAAAACGGGACTGGCT | CCAAACCAGCTCCTACTCCAG |
| BIRC3 | AGCTGCTATCCACATCAGACA | CGGCAGCATTAATCACAGGAG |
| UHMK1 | CCAGCCTATCACCTAAGAGAC | GGAGTGGGAAGCATGACCAGA |
| OIP5 | TCTGTGGTTCTTGTGGGATTC | TGGAAAGGCAGAAGTGACCTC |
| MMP9 | ACGCAGACATCGTCATCCAGT | GGACCACAACTCGTCATCGTC |
| GLO1 | ACCCCAGTACCAAGGATTTTCT | GGATTAGCGTCATTCCAAGAAC |
| TSC22D1 | GGGGAATGTCAGCAGTTTCAG | ACCACACTTGCACCAGAGGAG |
| PPP1R3C | CCCTGTCATTCCAACTGAGCA | CACCCCATCAGGCTTCCATTG |
| ARRDC3 | CAGCCATTTACCAAACACAGG | TTGCCATTCCACGTCTCTGTC |
| ARHGAP21 | CCCAGGAGATGTATCAGATTC | GATGGAGGACACAAGCAGTTC |
| GAS1 | GGCAAAGTCTTCAACGGGCTG | TCCTTGACCGACTCGCAGATG |
| SEMA3A | TCCAGAGGCGAAATGAAGAGC | ACCGCATGGCAGAGGTAATTG |
| GANAB | GCCAAGAGTTCCTGCTGCGTC | ATCACCACCCGCTCAATCCAG |
| OGFR | CAAGGTGAGGAAGCGGAGGAA | ACCCCGTTCTCACTGTGTCCA |
| TPM4 | TGAAAGAGGCTGAGACCCGTG | GTCTGATGTAAGCCCACGTTC |
| MESDC2  **Primer for ChIP-qPCR**  CCNB1  CCNE1  CCNE2  MCL1  BIRC3  UHMK1  Nanog  negative control  GAPDH  **Primer for cloning the coding sequence of WDR5** | AGAGGTTCATTGTGGGATCAG  AATTGGCCTTGGGAAACTGG  ACTGTTACCCAGGCTGGTCTC  CCTGGACGAGATCCTAGAGCA  CTTCCTTCAACCCCTGTGTTAGTC  GCCACAGTGACTTGCTTATTGG  ACACTGGGCCTGGAATGGTAGAT  GCTCGGTTTTCTAGTTCCCCA  GTAATCAGGAAACTGCATAC  A component from EZ-Magna ChIP A/G kit (Millipore), but no sequence of primer is provided.  ATGGCGACGGAGGAGAAGAAGC | CCAGAGTTACATCAGCACACC  CAGGGTCACACATTAGCAACG  CCCCATCTCTCACTTAAAGCC  CACCACACATGCACAGTTTCC  CTCTGTGCTTCCCTGAGACCTGAT  CCATTTCAACAGCAGAGACC  GCGGGGCCTAGAAGAGATTAG  ATCACCTTAGACCCACCCCTC  CTCAAGACTCAATAGTGATC  TTAGCAGTCACTCTTCCACAGTTTA |
| **Primer for cloning the coding sequence of Nanog** | ATGAGTGTGGATCCAGCTTGTCCC | TCACACGTCTTCAGGTTGCATGTT |

**Supplemental Figure 4**

Fig.2 A Fig.2 B


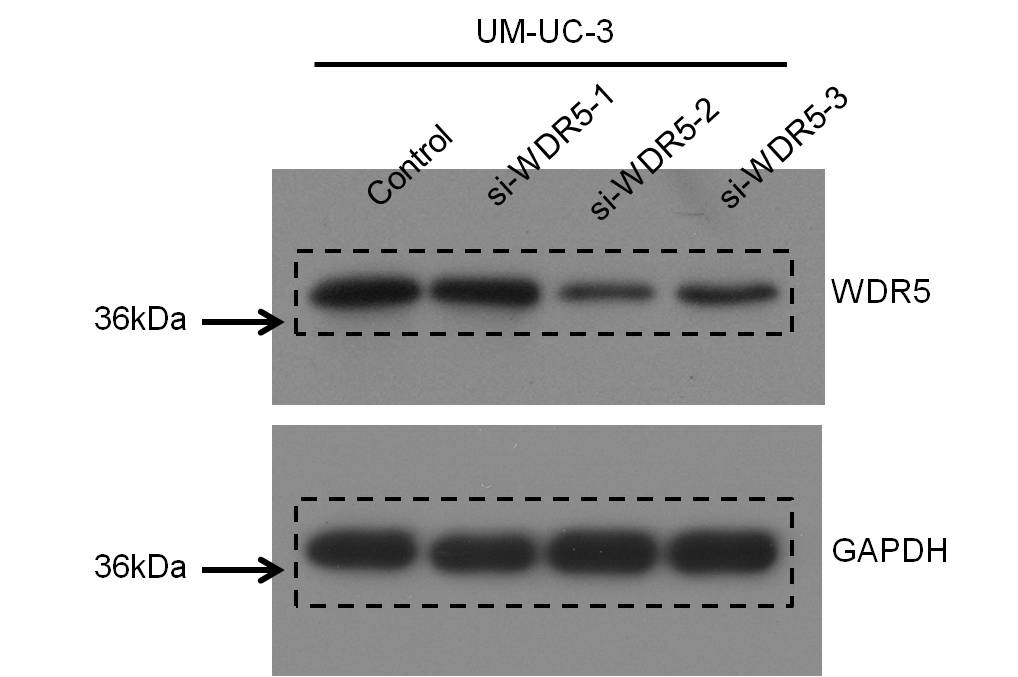

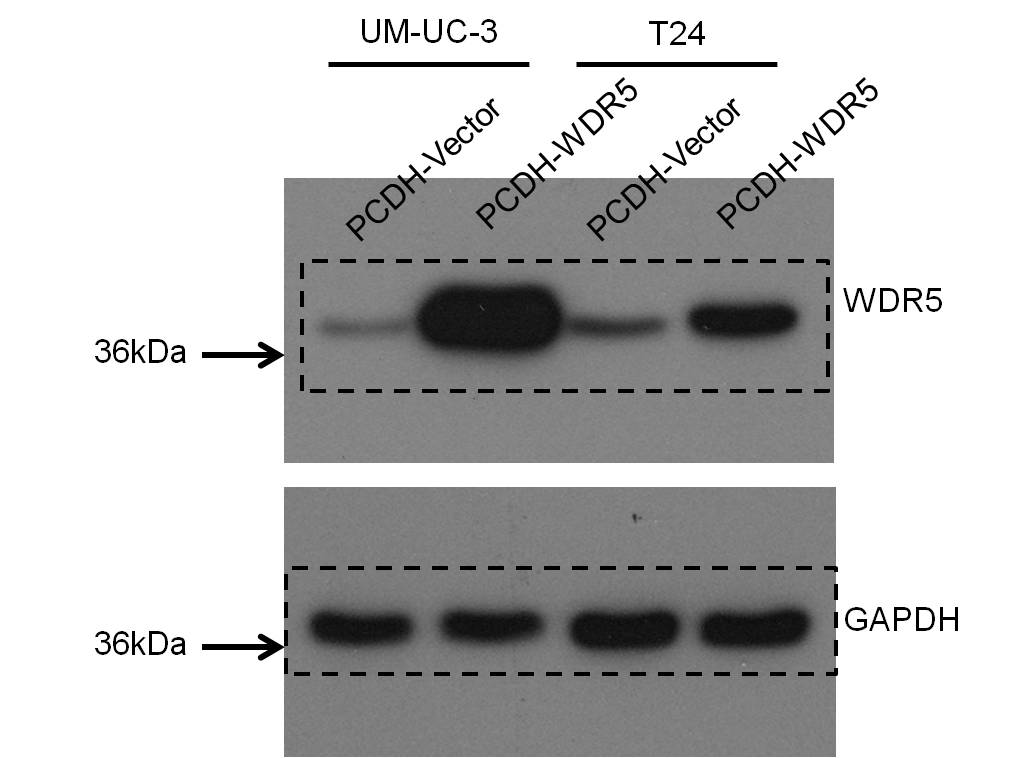


Fig.3D


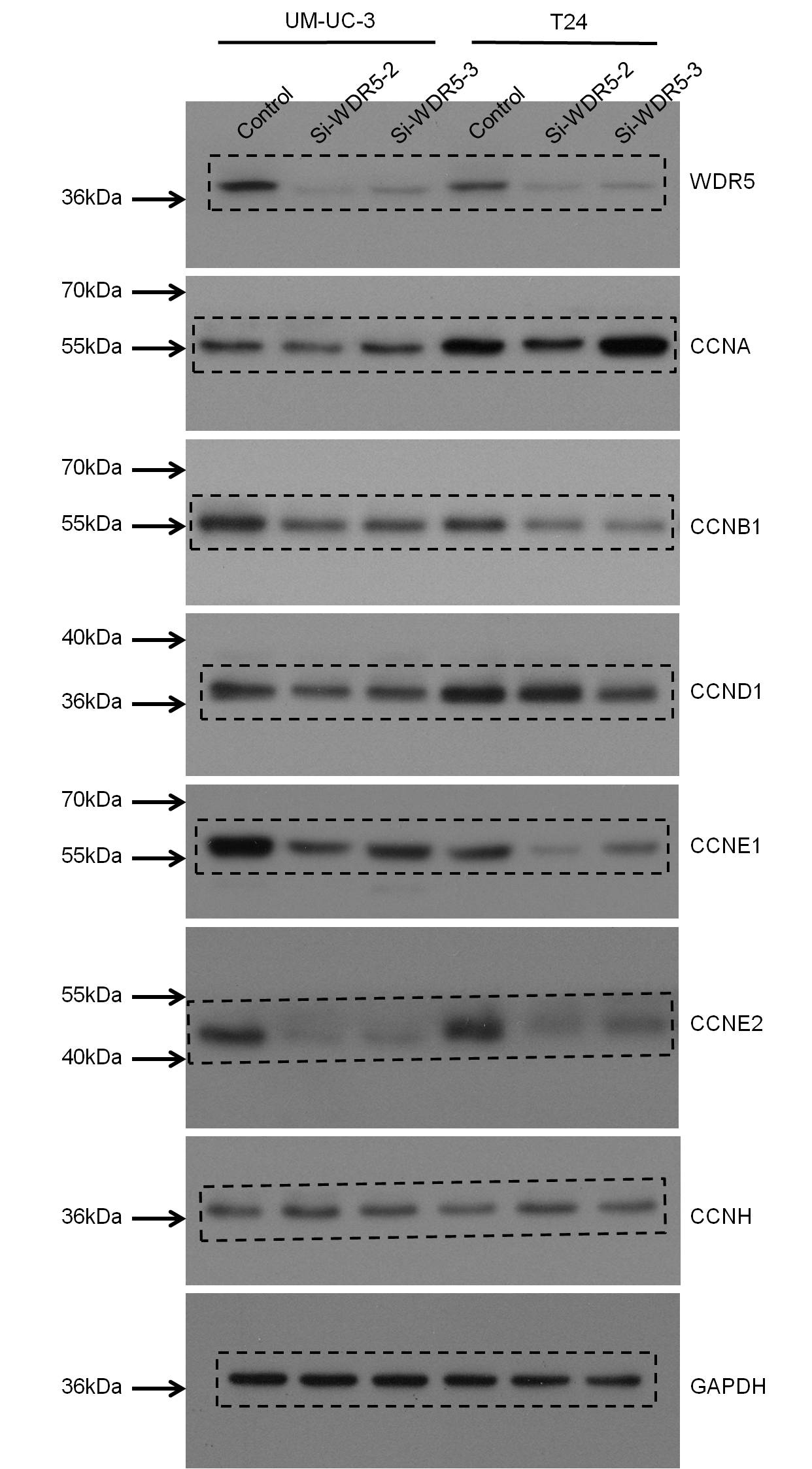


Fig.4A


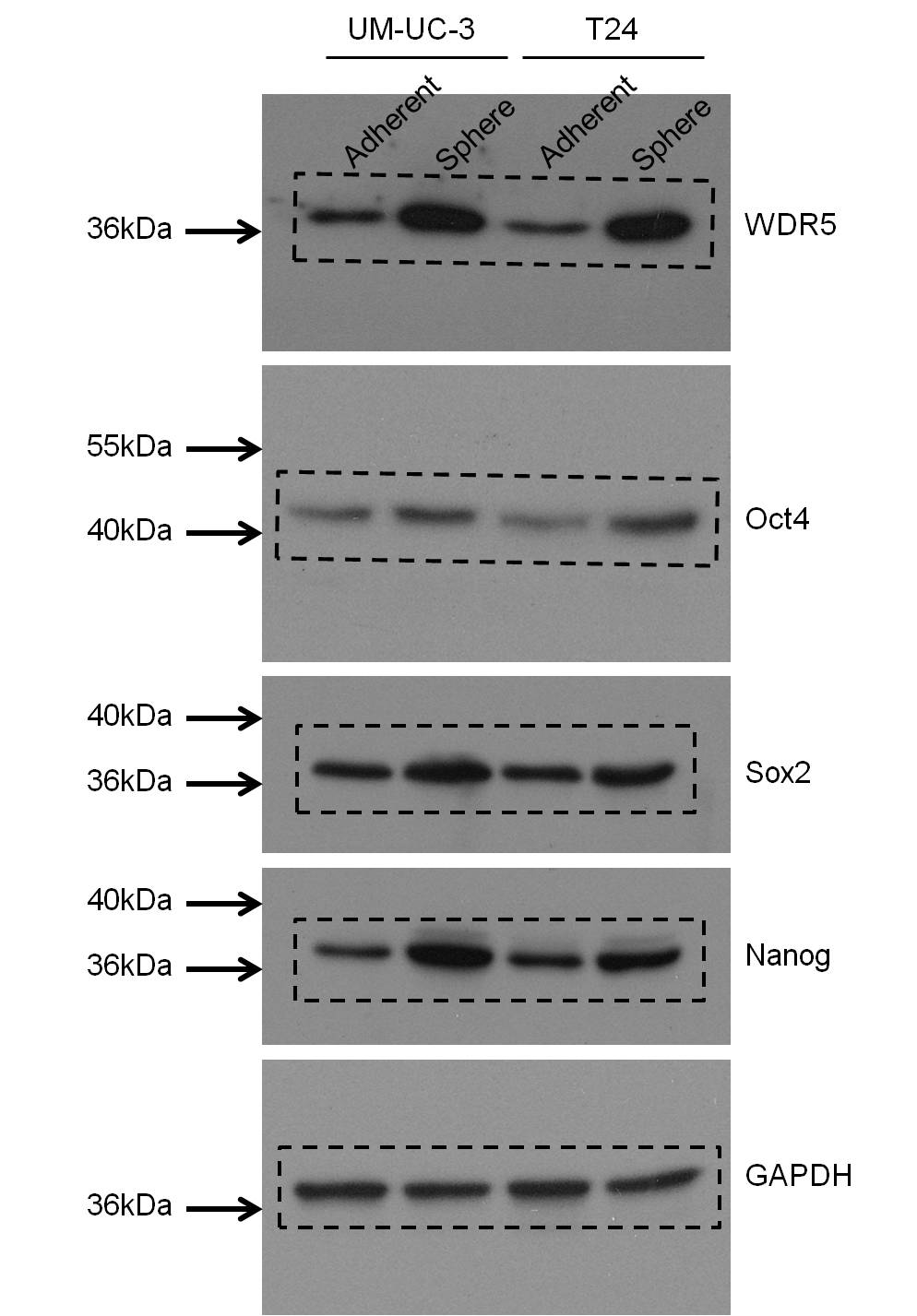


Fig.4G


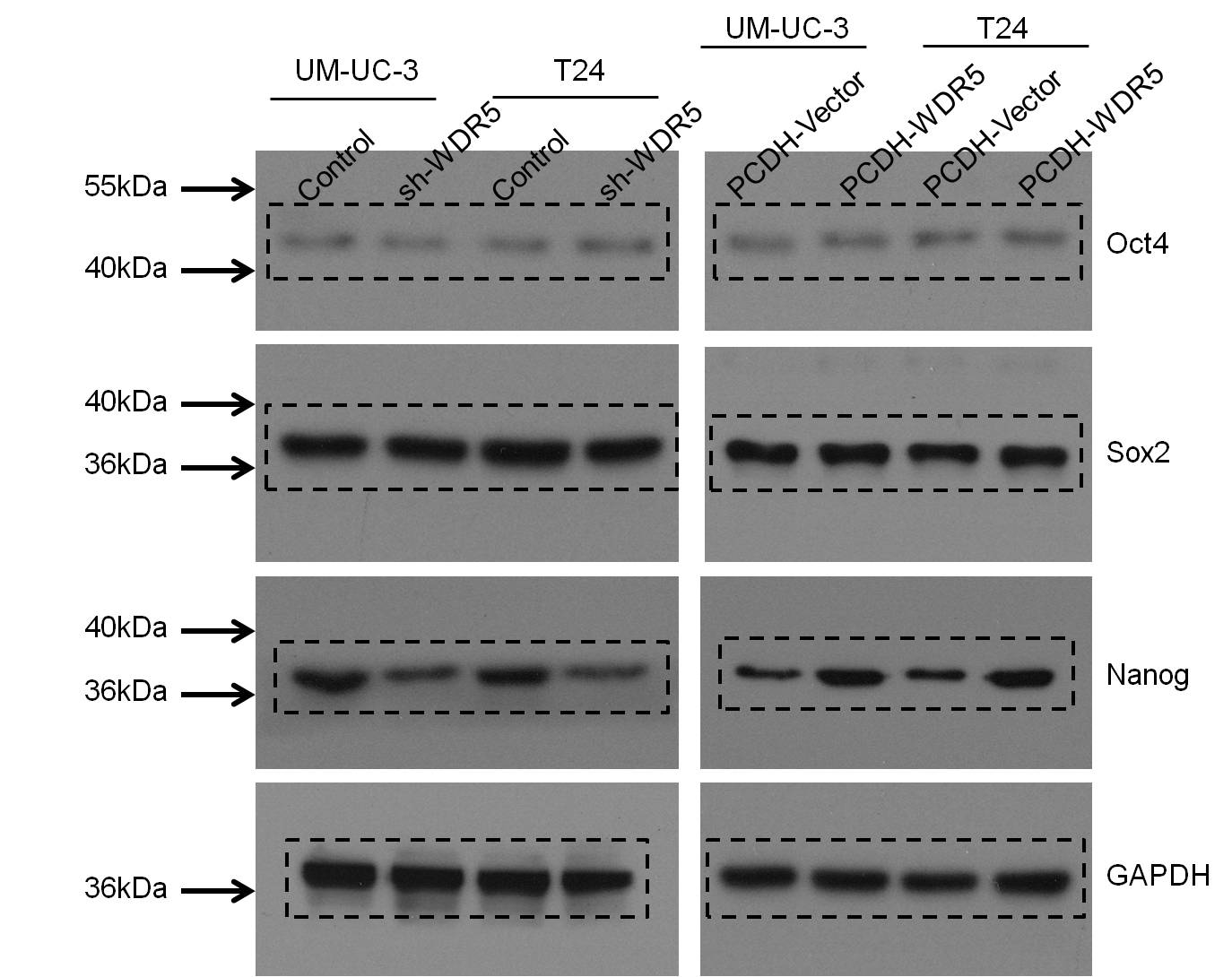


Fig.6G


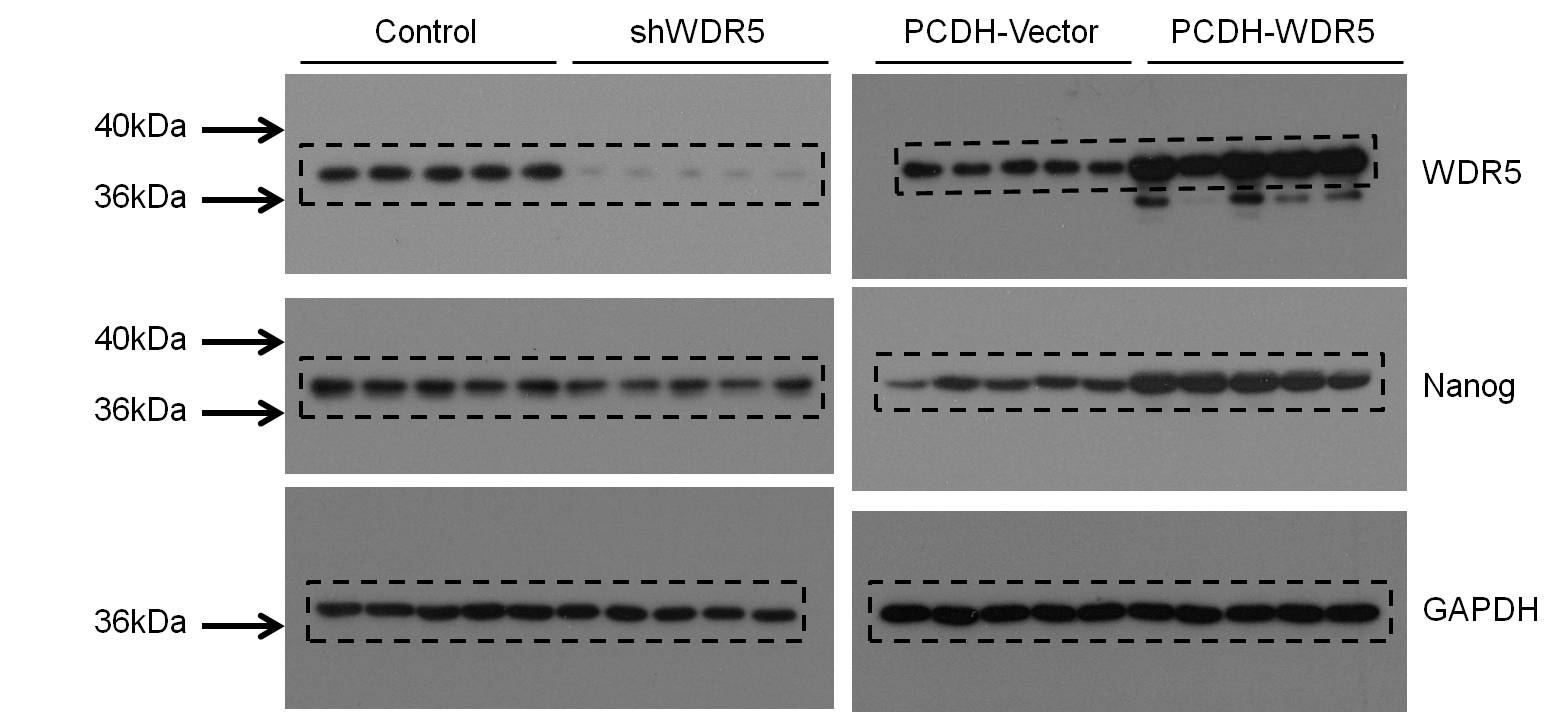


Fig.7D


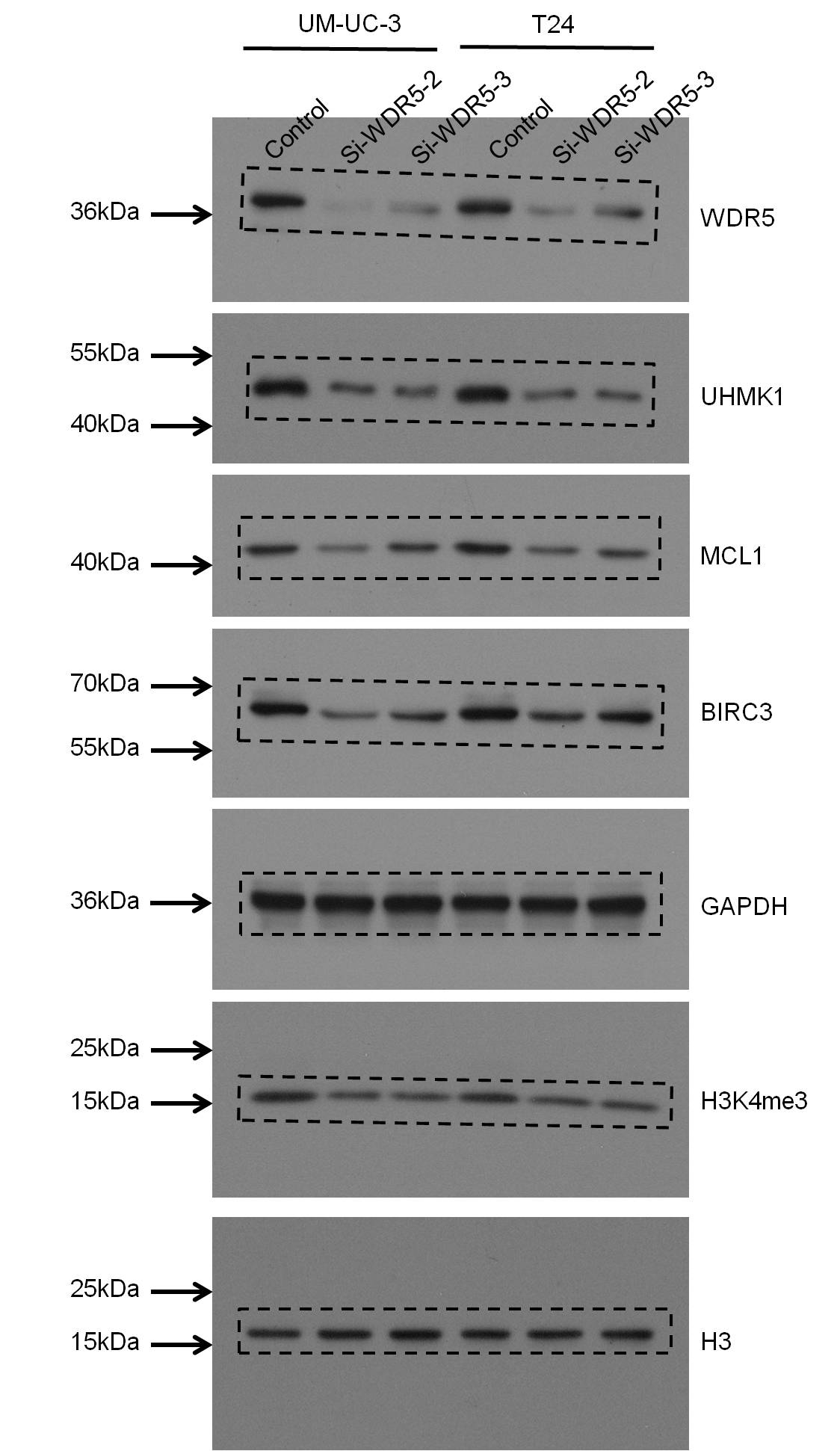


Supplemental Fig.2


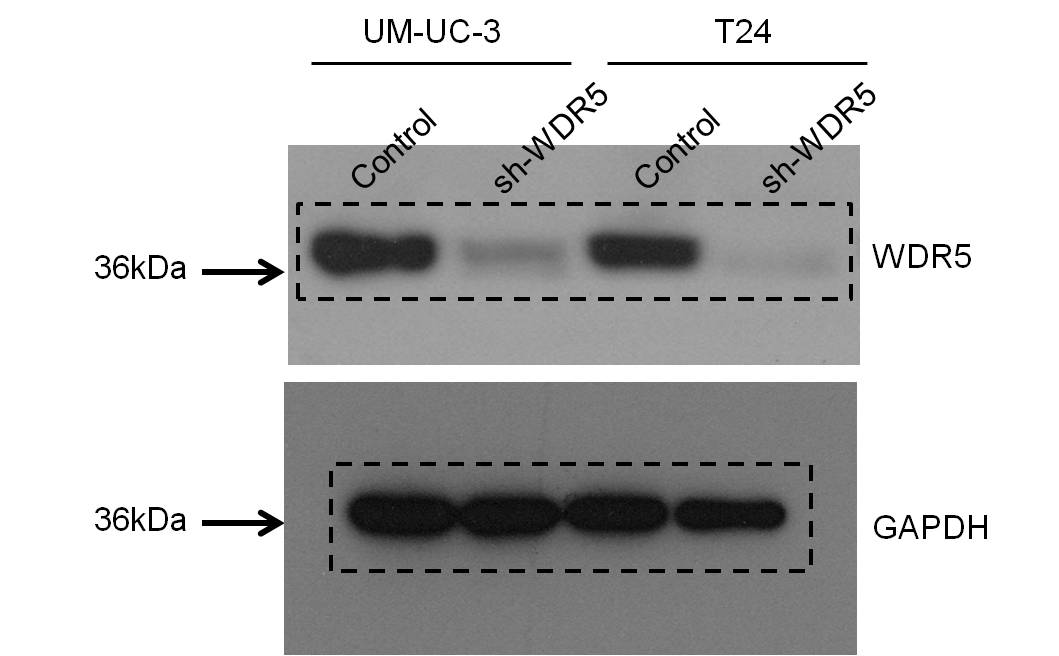


Supplemental Fig.3A


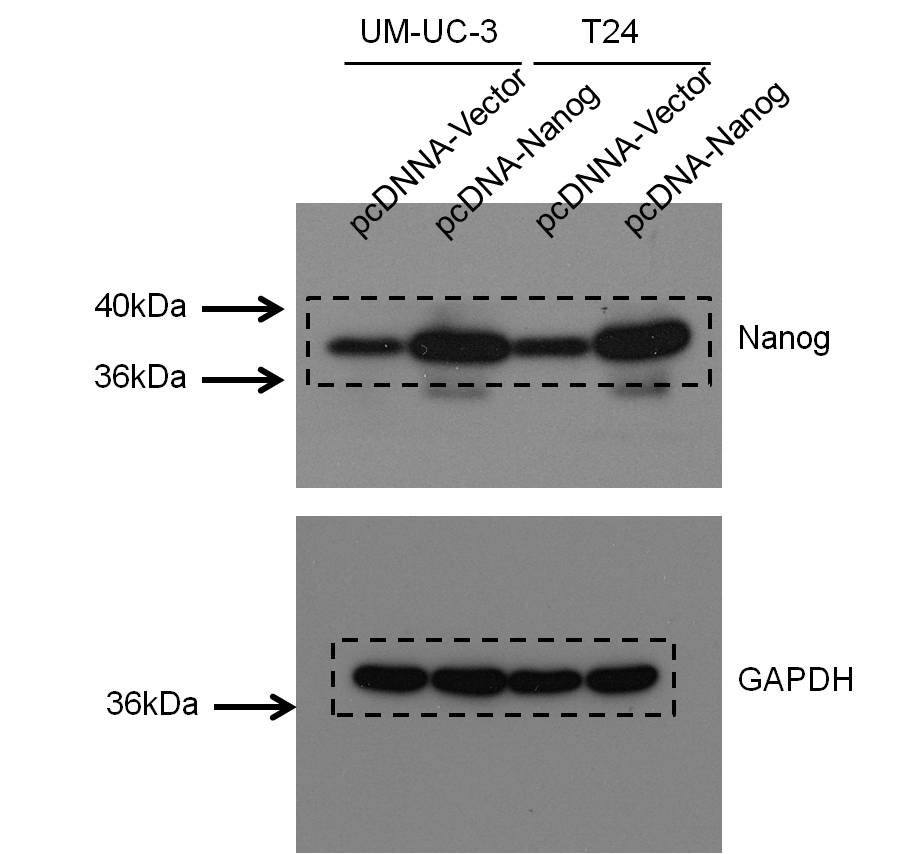


Supplemental Fig.6


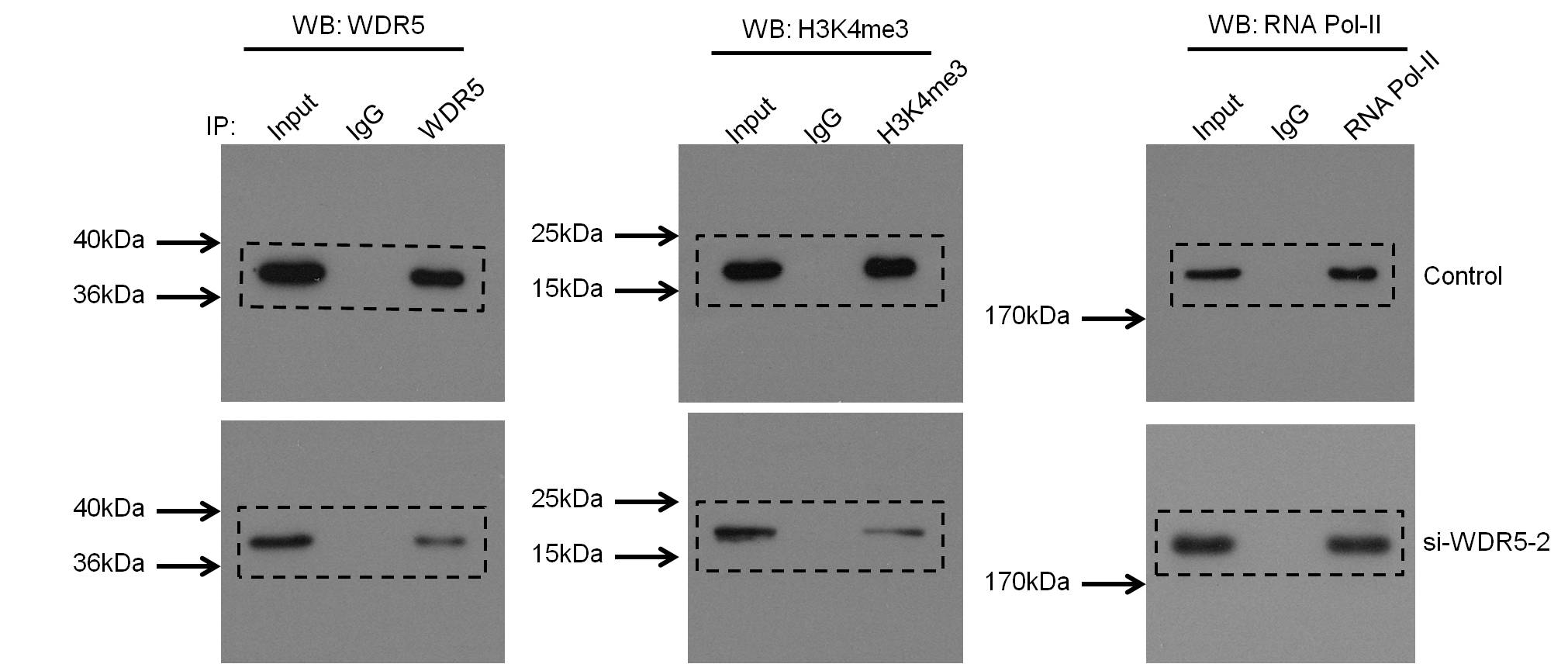

Supplement: Supplementary Information — Supplement [file srep08293-s1.doc]
